# Integrated SERS and Machine Learning Workflow for Nanoplastic Detection on a Plasmonic Membrane

**DOI:** 10.1021/acs.analchem.6c00075

**Published:** 2026-07-08

**Authors:** Amauri Horta-Velázquez, Erika Rodríguez-Sevilla, Angelica Hernandez-Rayas, Miguel A. Vallejo, Eden Morales-Narváez

**Affiliations:** † Centro de Investigaciones en Óptica (CIO), A. C., Loma del Bosque 115, Lomas del Campestre, León 37150, Guanajuato, Mexico; ‡ Biophotonic Nanosensors Laboratory, 73403 Universidad Nacional Autónoma de México, Centro de Física Aplicada y Tecnología Avanzada, Boulevard Juriquilla 3001, Querétaro 76230, Mexico; § División de Ciencias e Ingenierías, 14654 Universidad de Guanajuato, Colonia Lomas del Campestre, Campus León, Loma del Bosque 103, León 37150, Guanajuato, Mexico

## Abstract

Nanoplastics pose increasing health risks, necessitating sensitive and reliable detection methods. Surface-enhanced Raman spectroscopy (SERS) offers high sensitivity and molecular fingerprinting capabilities, but faces challenges in variability and data interpretation complexity, particularly for large analytes such as micro- and nanoplastics. Here, we propose an analytical framework that combines a SERS-active plasmonic membrane—nanopaper functionalized with gold nanorods— with a machine learning pipeline for the automated and semiquantitative detection of nanoplastics. The membrane format enables simultaneous collection and concentration of PMMA nanoplastics from aqueous samples. Our fully automated machine learning pipeline—integrating principal component analysis (PCA), Isolation Forests, K-means clustering, and an ExtraTrees classifier achieving 95% accuracy—enables interpretable, semiquantitative detection of PMMA nanoplastics without manual spectral analysis. Additionally, we incorporated an interpretability algorithm that identifies the vibrational modes driving the machine learning classification, yielding chemically validated and trustable predictions. After processing and interpreting the data with machine learning, semiquantification becomes feasible through peak-intensity calibration curves, with an estimated limit of detection of 0.02 μg mL^–1^. This workflow demonstrates the feasibility of integrating a SERS-active membrane with a machine learning workflow to streamline sampling, detection, and automated data interpretation. This marks an important advancement toward the development of field-deployable SERS-based platforms for easy and user-friendly nanoplastic monitoring.

## Graphical abstract

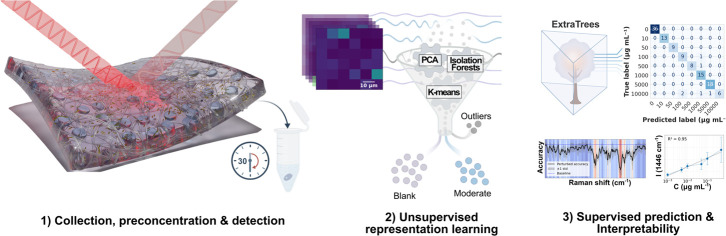

## Introduction

Plastics have improved our quality of life by providing affordable, lightweight, and durable goods that revolutionized industries such as packaging, healthcare, and technology. However, they also generated micro- and nanoplastics. Micro- and nanoplastics can be intentionally manufactured or created through the breakdown of larger plastics by mechanical stress, UV radiation, or high temperatures^1^ and dispersed by wind and water across ecosystems, accumulating in soil, waterways, and the atmosphere,^2^ posing risks to environmental and human health.

Plastic debris 1–1000 nm are typically considered nanoplastics, although a subcategory of submicron-plastics (100–1000) nm can be distinguished for consistency with nanomaterial classification.^3^ Their small size enables internalization by cells and subcellular structures, posing significant risks to living organisms.^4^ Growing evidence links nanoplastics exposure to serious conditions, including cancer,^5^ Parkinson’s disease,^6^ and other neurodegenerative, reproductive, and cardiovascular disorders,^4^ with nanoplastics detected in human brain, liver, and kidney tissues.^7^

In a typical sample, nanoplastics are expected to be present at low concentrations. In human tissue, nanoplastics have been found between 100 and 1000 μg g^–1^,^7^ while in freshwater and oceans, they range between 0.01 and 0.5 μg mL^–1^.^8^ Thus, highly sensitive detection techniques are needed for nanoplastic detection in real samples. Surface-enhanced Raman spectroscopy (SERS) is a suitable technique for its nondestructive nature, robustness in aqueous samples, high analytical sensitivity, and spectral fingerprinting ability. SERS detection is typically enabled by a substrate containing metallic nanostructures with a characteristic resonance wavelength, that is, the localized surface plasmon resonance (LSPR). These nanostructures enable the detection of analytes located near or between them (typically a few nanometers) when excited by a resonant laser. This light–matter interaction generates shifted wavelengths that are captured in a SERS spectrum, which is then analyzed for analyte identification and quantification.

Diverse SERS platforms with sufficient sensitivity to detect nanoplastics in real samples have been proposed (see Table 1). However, sample analysis typically requires treatment steps, such as microliter pipetting, mixing, filtration, centrifugation, preconcentration, or transfer to the SERS platform. Sample transfer is particularly problematic for nanoplastic (semi)­quantification since it can lead to significant errors due to sample loss.^9^

**Table 1:** Overview of Novel SERS Substrates Achieving Sensitivities Suitable for Nanoplastic Detection in Real-World Samples

SERS platform	nanoplastic (Size)	limit of detection (μg mL^–1^)	sample preparation steps	quantification capability	ref.
metal-phenolic networks and AuNPs	PS (1 μm, 500 nm, 50 nm)/PMMA (500 nm)/PE (740–4990 nm)/(PLA:250 nm)	0.1	mixing, centrifugation, and transfer	Yes	^10^
AgNPs and self-assembled PMMA film	PS (20, 100, 500, 1000 nm)/PET (70, 1000 nm)	10^–5^	mixing, addition of solvent, and sample transfer	Yes	^11^
hydrophobic AuNP film	PS (30–1000 nm)	0.03	preconcentration and transfer	No	^12^
microfluidic chip with Ag nanocavities	73 and 316 nm	10^–3^	microfluidic pumping	Yes	^13^
Klarite (commercial substrate)	PS (360,500, 1000 nm)/PMMA (360, 500 nm)	Single particle	filtration, preconcentration, and transfer	No	^14^

Abbreviations: PS = polystyrene, PMMA = poly­(methyl methacrylate), PE = polyethylene, PLA = polylactic acid, MOF = metal-organic frameworks, AuNP = gold nanoparticles, and AuNSs = gold nanostars.

A promising strategy to simplify nanoplastic detection is integrating filtration, preconcentration, and detection into SERS-active, plasmonic membranes. Thus, diverse SERS-active membranes fabricated using different substrates and metal nanoparticles have been proposed (Table 2). Among these substrates, nanopaper stands out for its high porosity, large surface area, optical transparency, and ease of functionalization.^15,16^ While recent studies have investigated bacterial nanocellulose for SERS-based detection of micro- and nanoplastics—specifically employing spherical silver nanoparticles or gold-core silver-shell nanoparticles to detect polyethylene and polystyrene— ^17,18^ functionalization with gold nanorods for nanoplastics detection remains unexplored.

**Table 2:** Overview of SERS-Active Membranes for Nanoplastic Detection

membrane material	metal nanoparticles	nanoplastic	limit of detection (μg mL^–1^)	sampling process	ref.
polyvinylidene fluoride	AgNPs	PS (30, 100n 500, 1000 nm)	0.001	filtration by suction	^20^
nitrocellulose	Au@AgNPs	PS (60 nm)	NR	drop cast and thermophoresis concentration	^21^
GO/Carbon nanotubes	AgNSs	PS (50, 100, 200 nm)	0.05	vacuum filtration	^22^
filter paper	AgNPs	PS (100, 300, 460, 600, 800 nm)	0.31	aggregation agent and syringe filtration	^23^
cellulose acetate	3D gold nanopockets	PS (1000 nm) and PE (1–4 μm)	2.5 and 5.3	syringe filtration	^19^
filter paper	AgNWs	PS (50, 100, 300, 500, 1000 nm)	10^–4^	syringe filtration	^9^
regenerated cellulose	AuNRs/AgNWs	PS (84, 444, 630 nm)	100	vacuum filtration	^24^
bacterial nanocellulose	Au-core/Ag-shell NPs	PE (65 nm) and PS (100 nm)	3.95 and 2.73	immersion	^18^

Abbreviations: PS = polystyrene, PE = polyethylene, AuNP = gold nanoparticles, AgNP = silver nanoparticles, AuNSs = gold nanostars, AgNWs = silver nanowires, AuNR = gold nanorods, NR = not reported.

Herein, we use a plasmonic membrane based on nanopaper functionalized with gold nanorods. This membrane is low-cost (≈$0.12USD, Table S1), fabricated in a single immersion step, and disposable by combustion (Supplementary Video S1). When immersed in liquid samples, the nanopaper can adsorb and retain nanoplastics, functioning as a membrane-like support that creates an extended interaction volume via multiple internal reflections, along with a uniform adsorption of gold nanorods.

SERS-based nanoplastics detection requires trained professionals for complex data acquisition and analysis. Typically, nanoplastic (semi)­quantification using SERS membranes requires examining the spatial SERS signal distribution across the membrane through surface maps,^9,19^ involving manual review of large spectral collections to identify plastic-related peaks and outliers, a tedious, error-prone process.

To advance nanoplastics (semi)­quantification toward real-world application, automated data analysis is essential.^25^ Therefore, we propose an integrated analytical framework combining a SERS-active membrane with a robust, fully automated machine-learning pipeline. This system addresses the spatial heterogeneity inherent in micro- and nanoplastic-derived SERS signals, ensuring reliable semiquantification. Our framework incorporates an interpretability algorithm tracing model predictions to specific SERS peaks and vibrational modes, ensuring that the model relies on chemically meaningful spectral information for transparent and trustworthy predictions.

After collecting nanoplastics by simple immersion, the membrane is directly analyzed using our machine-learning-assisted SERS approach to detect and semiquantify nanoplastics. This effectively combines collection, preconcentration, and accurate detection into a single platform, see Scheme 1. We validated this workflow by using spiked samples with PMMA nanoplastics as a model analyte. This setup isolates the analytical contributions of both the membrane and the nanoplastics, allowing us to evaluate and establish the machine-learning-based workflow for the automated, semiquantitative nanoplastic detection.

**Scheme 1:**
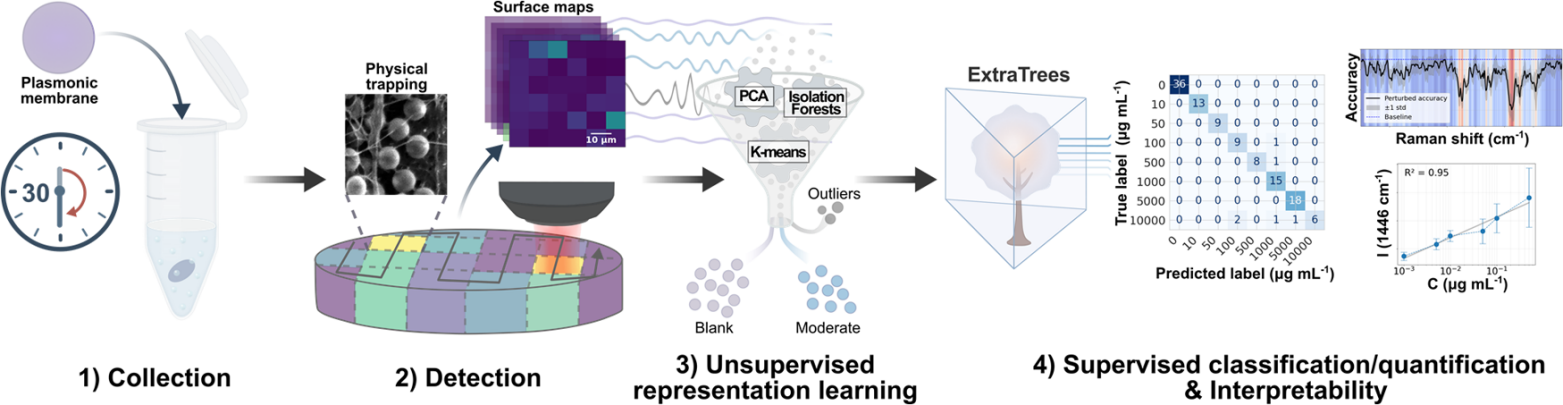
Integrated Collection, Mapping, and Machine-Learning Analysis of Nanoplastics Using a Plasmonic Nanopaper Membrane. (Left) The Plasmonic Membrane Is Immersed in a Liquid Sample Containing Nanoplastics, Enabling Simultaneous Collection and Retention of Nanoplastics within Its Porous Structure; (Center) the Membrane Is then Analyzed by SERS through Spatially Resolved Surface Maps Exhibiting Strong Spatial Variability Due to Heterogenous Enhancement in the Large-Particle Regime; and (Right) the Surface Map Is Processed Using an Automated Machine Learning Pipeline Combining PCA for Dimensionality Reduction and Visualization, Isolation Forests for Outlier Removal, and K-Means Clustering to Isolate Spectra with Moderate Enhancement. A Comprehensive Set of Supervised Machine Learning Models Were Evaluated, from Which ExtraTrees Stand Out with a 95% Cross-Validated Accuracy for Classifying Spectra into Concentration Levels. Interpretability Analysis Validates that the Machine Learning Model Relies on Chemically Meaningful Features from Nanoplastics, Enabling Extraction of the Most Informative Raman Shifts to Build Calibration Curves for Semiquantitative Detection. This Workflow Combines Sampling, Pre-concentration, Detection, and Automated Interpretation in a Single Platform

## Results and Discussion

### Leveraging Nanopaper for Enhanced Interaction with SERS Excitation Volumes

Confocal Raman microscopes produce excitation volumes that depend on both measurement conditions and optical properties of the sample. Consequently, the diffraction-limited resolution of ≈1 μm is rarely reached. In practical scenarios, significant out-of-focus contributions to the Raman signal extend over much longer ranges, easily reaching several tens of microns, particularly in transparent and scattering samples.^25^

A three-dimensional (3D) SERS substrate that interacts with the huge excitation volumes generated in Raman microscopes—compared to nanoscale hot spots—offers advantages for (semi)-quantification. For example, collecting signals from larger excitation volumes increases laser interaction probability with analyte–hot spot interfaces, useful for low concentrations and trace detection. 3D SERS substrates may also enhance sensitivity by increasing the number of analyte–metal nanoparticle interfaces contributing to Raman signal intensity. Additionally, integrating signals from a larger volume of uniformly distributed nanostructures can improve reproducibility by averaging local variations.^26,27^

Nanopaper (nanostructured paper-like cellulosic material) exhibits high optical transparency and undergoes multiple internal light-scattering events due to numerous micro- and nanoscale refractive index inhomogeneities.^28^ The extent of the light scattering depends on the nanostructure size and morphology—e.g., cellulose nanocrystals or nanofibers—as well as on the density and porosity of the nanopaper, which determines its transparency.^29,30^ Consequently, nanopapers range from fully transparent (low-scattering) to completely opaque white (highly scattering).

For SERS, nanopapers with an intermediate semitransparent state, exhibiting partial scattering, are likely optimal. Semitransparency increases a laser–analyte interaction through multiple scattering and reflection events while preserving sufficient transparency for laser penetration and efficient light collection. Bacterial cellulose nanopaper achieves this balance, displaying a frosted, hazy appearance due to its 3D nanofibrillated structure with an extinction spectrum dominated by Rayleigh scattering (Figure 1a and c). Remarkably, nanopaper creates this highly scattering medium with only a few micrometers thick, in this case ≈16 μm.^30,31^

**Figure 1:**
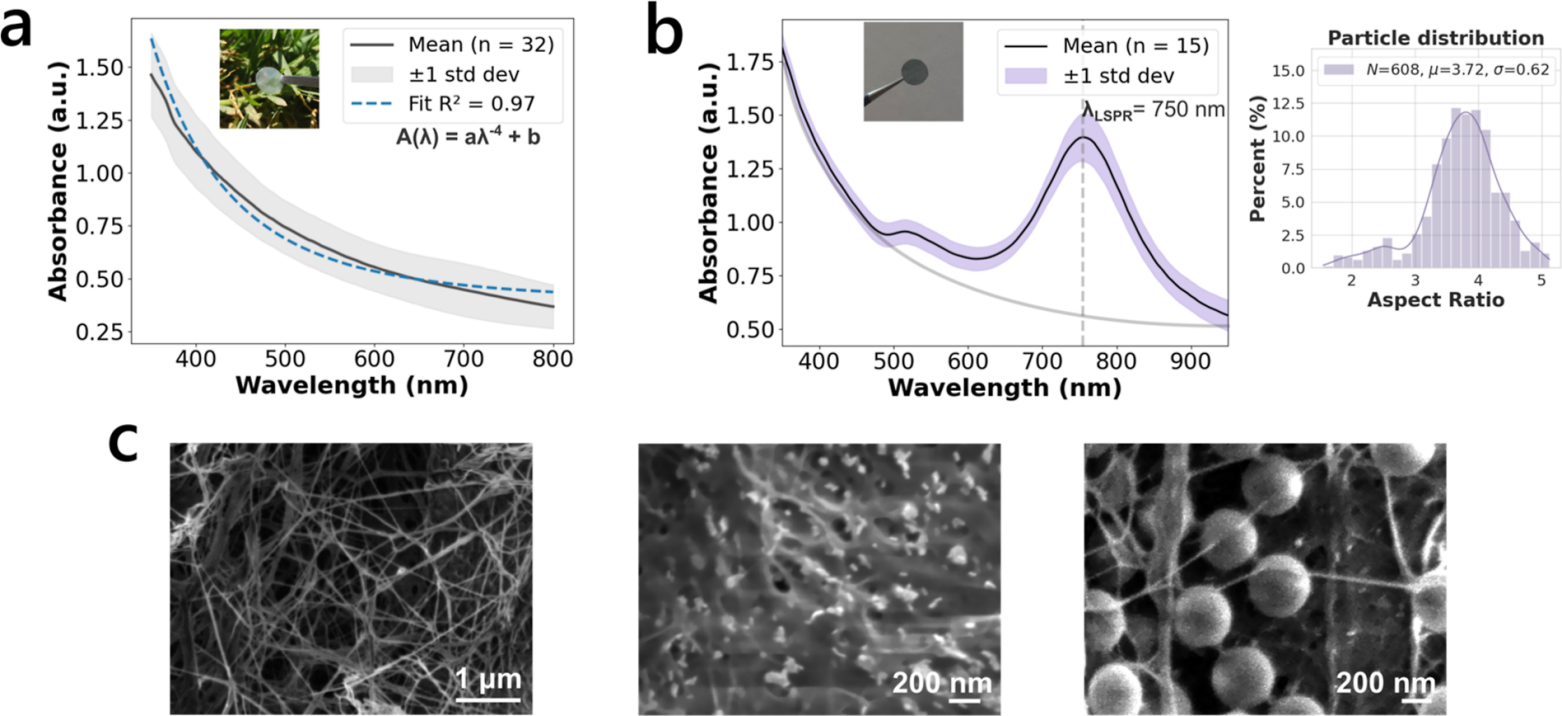
Optical and structural characteristics of bacterial cellulose nanopaper and the plasmonic membrane. **a**. Absorption spectrum of pure nanopaper and its fitting function *A­(*λ*)* = *a*λ^–4^ + *b*. Agreement with the model indicates light attenuation is dominated by Rayleigh scattering, with coefficients *a* and *b* reflecting scattering strength and a wavelength-independent baseline from residual optical losses. **b**. Absorption spectrum of gold nanorod-functionalized nanopaper (left) and nanorod aspect ratio distribution (right). Gold nanorods showed a length of c.a. 40 ± 7 nm and a width of c.a. 11 ±1 nm. The aspect ratio distribution explains the localized plasmon resonance (LSPR) position and band broadening in the UV–vis–NIR absorption spectrum (left; black line). Preservation of the scattering baseline (gray line; left) confirms plasmonic functionality is integrated without altering the multiple-scattering regime of the nanopaper. **c**. SEM images of pure nanopaper (left), the plasmonic membrane with gold nanorods adsorbed on the surface (center), and the membrane after immersion in a nanoplastic-spiked sample at 1000 μg mL^–1^ (right). The images highlight the fibrillar network of nanopaper responsible for multiple internal scattering, gold nanorod adsorption, and retention of PMMA nanoplastics within the porous structure.

The nanofibrillated structure of bacterial cellulose nanopaper gives it a membrane-like architecture. However, rather than having a single, well-defined pore size, nanopaper is a hierarchical porous membrane with pore sizes across multiple length scales. These pore sizes extend from mesoporosity to macroporosity, ranging from tens of nanometers to micrometers, resulting in a specific surface area of 10–100 m^2^ g^–1^.^32–34^ This broad structural distribution makes nanopaper a suitable high-surface-area scaffold for the adsorption, entrapment, and filtration of various particle sizes.

For functionalization, different nanomaterials can be loaded into the nanopaper via simple immersion, such as gold and silver nanoparticles, transforming it into a plasmonic membrane.^31,35,36^ Here, we functionalized nanopaper with gold nanorods for their balance between electromagnetic enhancement and signal reproducibility.^37^ The employed AuNRs showed a length of 40 ± 7 nm and a width of 11 ± 1 nm (Figure S2). This functionalization introduces a plasmonic response suitable for SERS-based detection while preserving the multiple scattering regime of the nanopaper (Figure 1b).

The plasmonic membrane exhibits excellent structural stability in aqueous samples, both in the short term (0.5 to 60 min) and long-term (24 h). Following immersion, no significant detachment of nanorods occurs, and the multiple scattering regime of the nanopaper is maintained (Figure S1). Effective loading and stable adsorption of the nanorods into the nanocellulosic matrix are facilitated by electrostatic interactions between the partially negatively charged bacterial nanocellulose and the positively charged cetyltrimethylammonium bromide (CTAB) bilayer stabilizing the gold nanorods. These interactions are reinforced by hydrogen bonding between the hydroxyl groups of nanocellulose and CTAB.^38,39^

We evaluated batch-to-batch reproducibility, including gold nanorod morphology, absorbance spectrum, and SERS background signal, across three independent batches of gold nanorod synthesis and membrane fabrication (Figure S2). This evaluation showed consistency in gold nanorod incorporation, optical response, and background signal, where the most intense background band at 1615 cm^–1^ showed an interbatch relative standard deviation (RSD) of 12%. Overall, nanopaper is an excellent choice for constructing plasmonic membranes capable of interacting with the extended 3D excitation volume of SERS measurements.

### Overcoming Hot Spot Inaccessibility: Nanoplastic Trapping in Plasmonic Membranes

In a typical SERS substrate, the target analyte must be in privileged sites called hot spots, formed in the nanogap (≈2–10 nm) between nanoparticles.^40^ Thus, nanoplastics larger than 10 nm cannot access them, requiring alternative detection strategies. Plasmonic membranes also offer advantages in this context.

When immersed in a nanoplastic-spiked solution, the plasmonic membrane can effectively trap PMMA nanoplastics within its porous structure (Figure 1C). This configuration provides a volumetric distribution of nanoparticles near the nanoplastics, generating moderate but numerous enhancement zones around and inside the nanoplastics, supporting their detection by SERS.^41^

In this case, PMMA nanoplastics have a nominal size of 500 nm, so retention primarily occurs through physical entrapment within voids of the membrane of similar or larger sizes (Figures 1c and S3). However, bacterial cellulose nanopaper is a hierarchical porous material with pore sizes ranging from tens of nanometers to micrometers,^32–34^ as observed via electron microscopy (Figure S4). Other systems based on bacterial cellulose nanopaper can retain particles in the sub-100 nm range, including 65 nm polyethylene nanoplastics.^15,16,18^ Thus, while size-dependent capture efficiency requires evaluation, detection of smaller nanoplastics is feasible.

### Spectral Variability in Nanoplastic SERS Detection

Since bacterial cellulose nanopaper and the plasmonic membrane (nanopaper with gold nanorods) have their own Raman features, the characteristic peaks of PMMA must be distinguishable from the substrate background. Figure S5a shows the Raman spectra of pure bacterial cellulose nanopaper, the plasmonic membrane, and PMMA nanoplastics deposited on a nanopaper substrate (under non-SERS conditions). The characteristic PMMA peaks, located around 635, 693, 1268, 1448, and 1730 cm^–1^, associated with the C–C–O stretching, C–O–C stretching, O–CH_3_ bending, and C=O stretching modes,^14,42,43^ are visible and differentiable from both nanocellulose and the plasmonic membrane. Peak assignments of PMMA and the plasmonic membrane are provided in Tables S2 and S3, respectively.

To assess plasmonic membrane semiquantification capability, membrane samples were immersed in spiked dilutions containing varying concentrations of PMMA nanospheres, ranging from 10 to 10,000 μg mL^–1^. Average SERS spectra for 500 nm PMMA nanoplastics at different concentrations, measured at multiple points across the membrane, are shown in Figure S5-b. This approach does not reveal a clear correlation between PMMA peak intensity and nanoplastic concentration, mainly due to the high signal variability across the membrane (Figure S5-c).

A comprehensive approach for nanoplastics semiquantification uses surface mappings to probe the spatial distribution of nanoplastics across the membrane, typically point by point within a user-defined area.^9,19^ These mappings must be preprocessed as SERS spectra intensity at any scanning point varies depending on 1) the number of enhancement zones within the excitation volume—defined as the number of interfaces between metal nanoparticles and nanoplastics—and 2) the enhancement generated by each interface.^44^

Assuming a uniform nanoparticle density, the number of enhancement zones is proportional to the number of nanoplastics trapped in the excitation volume. Thus, SERS spectra intensity correlates with nanoplastic concentration, enabling (semi)­quantitative analysis. Meanwhile, the enhancement magnitude at each metal nanoparticle–nanoplastic interface depends on the distance between the nanoparticle and the nanoplastics. Additionally, for anisotropic nanoparticles, such as gold nanorods, the SERS intensity and shape depend on nanoparticle orientation relative to the nanoplastic surface,^45^ see Scheme 2.

**Scheme 2:**
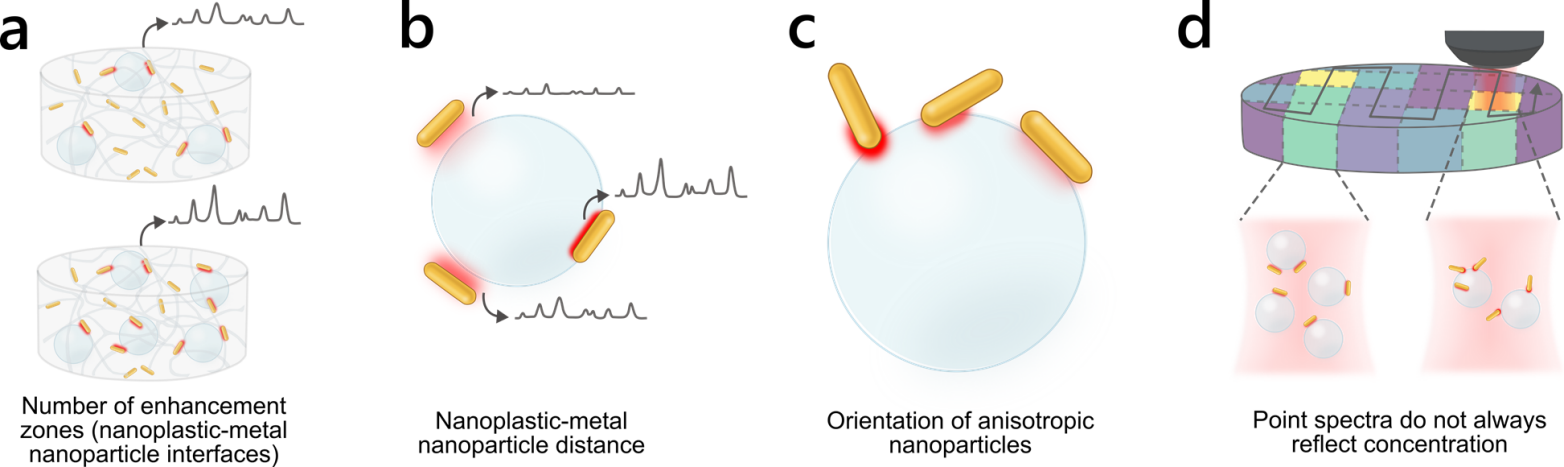
Factors Influencing Spatial Variability in SERS Intensity across Surface Mappings for Large Particle Targets, Such as Nanoplastics. **a** The Number of Enhancement Zones within the Excitation Volume. Samples with More Nanoplastics Generate More Metal Nanoparticle–Nanoplastic Interfaces (Considering a Uniform Metal Nanoparticle Density), Yielding Stronger Spectra. **b** SERS Intensity at Each Interface Depends Strongly on Nanoplastics–Metal Nanoparticle Distance. **c** For Anisotropic Nanoparticles, Such as Gold Nanorods, the Relative Orientation Strongly Modulates SERS Intensity. The Strongest Enhancement Occurs when Nanoparticle Tips Lie Close to the Nanoplastic Surface. **d** Due to these Combined Factors, Regions with Few Nanoplastics Can Sometimes Produce High-Intensity Spectra Comparable to those from Samples with Concentrations Several Orders of Magnitude Higher, if Local Distances and Orientations Favor Stronger Enhancement. This Explains the Substantial Spatial Variability in the Large Particle Regime Even for SERS Substrates with Excellent Reproducibility for Small Molecules and Why Point-Level Spectral Intensity Does Not Reliably Reflect Bulk Nanoplastic Concentration

Consequently, even with uniform metal nanoparticle distribution across the membrane, local inhomogeneities in trapped nanoplastics distribution as well as variations in metal nanoparticle–nanoplastic distances and orientations lead to fluctuations in SERS spectra intensity and shape across mapping scanning. Hence, the spectral intensity at each point does not always reflect the nanoplastic concentration of the sample.

Even in SERS substrates with excellent reproducibility for small analytes, reproducibility is significantly reduced for larger particles like nanoplastics.^41^ Surface mappings showed substantial variability across points on the membrane (Figure 2a), and low-concentration samples occasionally generated high-intensity spectra comparable to those from samples with concentrations 2 orders of magnitude higher (Figure S6). This spatial heterogeneity limits semiquantification by the conventional strategy of measuring spectra at different points and computing the average to build peak intensity concentration correlations.

**Figure 2:**
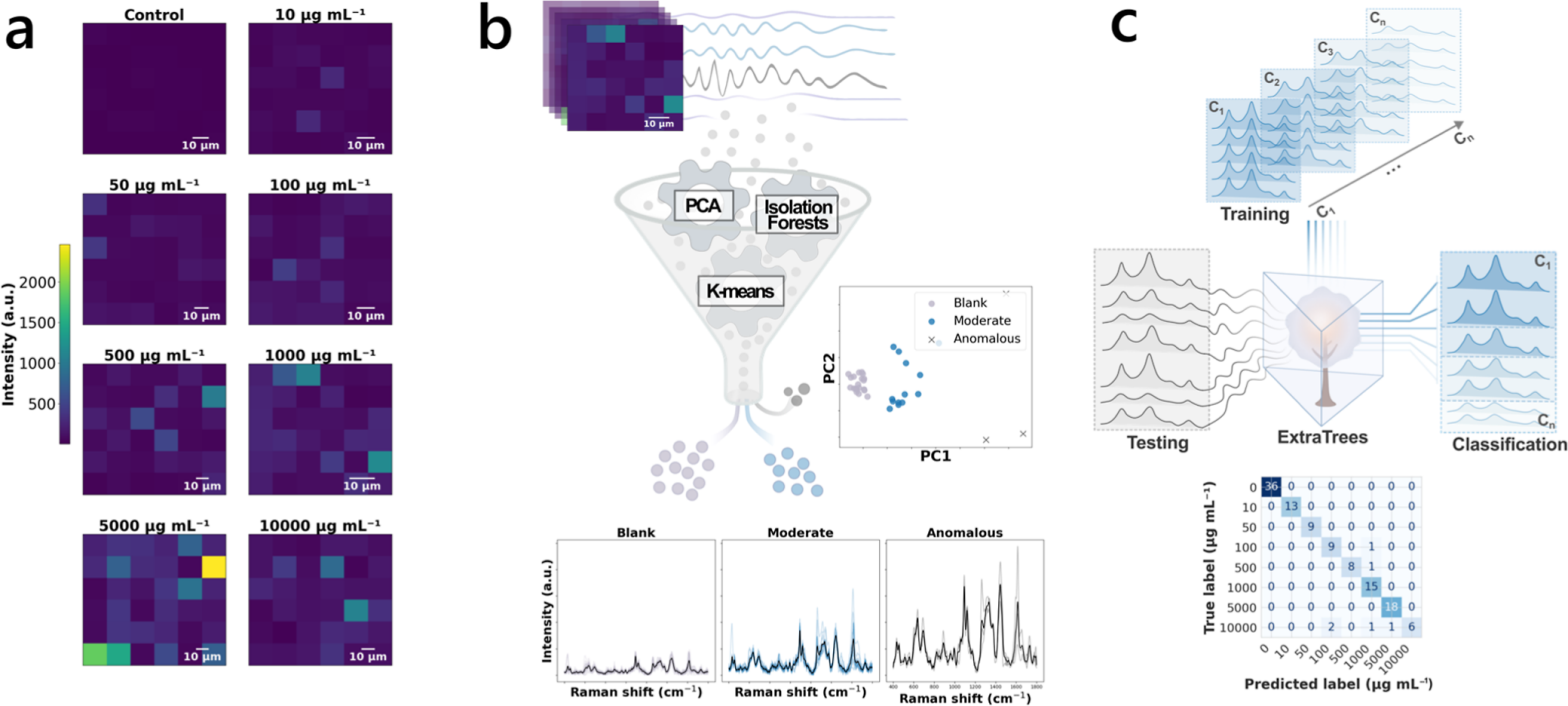
Spatial heterogeneity in SERS nanoplastic maps and unsupervised workflow for spectrum selection prior to semiquantification. **a** SERS surface mappings of PMMA nanoplastics (10–10,000 μg mL^–1^) acquired on the plasmonic membrane showing strong point-to-point variability. **b** Unsupervised pipeline combining PCA, Isolation Forests, and K-means for spectra clustering into blank, moderate-enhancement, and anomalous categories. Only moderate-enhancement spectra are retained for downstream semiquantification. **c** Confusion matrix of the ExtraTrees classifier, demonstrating semiquantitative assignment of nanoplastic-containing samples to discrete concentration levels.

### Surface Maps Processing via Unsupervised Learning

Integrating machine learning with SERS has been appointed as a promising and versatile strategy across several fields,^46,47^ including nanoplastic detection.^48^ Table 3 summarizes the relevant literature on this topic. Most methodologies use machine learning for plastic type identification or building regression models.^49–52^ While outlier detection using Isolation Forests has been explored,^10^ it is primarily applied to identify nonplastic, negative spectra and requires prefiltering of the SERS data sets to remove the inherent high spatial heterogeneity.

**Table 3:** Overview of Machine Learning-Assisted SERS Strategies Implemented in Nanoplastic Detection

SERS substrate	nanoplastic (size)	machine learning model	machine learning tasks	ref.
Metal–phenolic networks and AuNPs	PS (1 μm, 500 nm, 50 nm)/PMMA (500 nm)/PE (740–4990 nm)/(PLA:250 nm)	PCA, isolation forests, and classifiers (SVM, KNN, RF, and K-means)	detection of nonplastic spectra and plastic identification^a^	^10^
hydrophobic CuO@Ag nanowires	PS (50 nm)	PLSR and SVR	regression (quantification)	^52^
Ag nanodendrites	PS (67 and 470 nm)	CNN	plastic identification	^50^
superhydrophobic Ag-PDMS-Al surface	PS, PET, and PP (50 nm all)	t-SNE, CNN and classifiers (KNN, DT, RF, and SVM)	plastic identification	^49^
AgNPs in a Capillary tube	PS (70 and 300 nm)	CNN	plastic identification	^51^
AuNR-functionalized nanopaper	PMMA (500 nm)	PCA, Isolation Forests, K-means, and 11 classifiers^b^	automated map processing, semiquantification, and model interpretation	this work

a The input data set is a prefiltered spectra set collected on the coffee ring.

b Consult Table S4.

Abbreviations: PS = polystyrene, PMMA = poly­(methyl methacrylate), PE = polyethylene, PLA = polylactic acid, AuNP = gold nanoparticles, AuNR = gold nanorods, PCA = principal component analysis, SVM = support vector machines, SVR = support vector regressor, KNN = k-nearest neighbors, RF = random forests, DT = decision trees, PLSR = partial least-squares regression, CNN = convolutional neural networks.

Here, we propose a fully automated, end-to-end machine-learning pipeline that directly accepts SERS surface mappings as input. This pipeline autonomously clusters data points into blank, moderate enhancement, and anomalous regions, delivering interpretable semiquantification results. Specifically, we implemented a fully unsupervised workflow combining principal component analysis (PCA) for dimension reduction, isolation forest for outlier detection, and K-means for clustering spectra into meaningful categories, followed by semiquantification using a machine learning classifier (Figure 2).

PCA reduces the high dimensionality of SERS spectra by transforming thousands of Raman shifts into a lower dimensional space, typically comprising a few uncorrelated principal components. This step preserves the key variance patterns associated with the SERS enhancement while filtering out minor, irrelevant fluctuations. Spectra with similar variability patterns, such as shared spectral features or enhancement profiles, are positioned closer together, while spectra with distinct patterns lie farther apart, improving the visualization and facilitating subsequent outlier detection and clustering.

In the PCA-transformed space, an isolation forest detects outliers by identifying spectra based on how quickly they separate from the rest of the data set using random splits of the PC features. Spectra with intense or inconsistent profiles, which are common in the highest enhancement scenarios, are efficiently flagged as anomalies.

After outlier removal, K-means clustering groups the remaining spectra based on the PCA-transformed space. Unlike supervised algorithms such as logistic regression, K-means does not require predefined labels or a training phase; it requires specifying only the number of expected clusters. K-means identifies natural groups based on intrinsic variability patterns, enabling the data-driven separation of spectra into meaningful categories.

The PCA + isolation forest + K-means workflow automatically assigns each spectrum from the surface mappings into categories that represent an average of the nanoplastic–plasmonic membrane interactions under the measuring region (Figure 2b): 1) blank spectra, corresponding to regions where nanoplastics were absent or not enhanced (i.e., not located near a gold nanorod), 2) spectra exhibiting moderate enhancement, corresponding to regions where the nanoplastics are sufficiently close to some gold nanorods, and 3) anomalous spectra, arising from regions of high enhancement, produced in regions of exceptionally strong enhancement, typically due to a very close proximity between nanoplastics and metal nanoparticles or favorable orientations, such as nanoplastics located near gold nanorod tips. These anomalous spectra exhibit higher intensities, greater variability, and sometimes altered spectral shapes.

Anomalous spectra were identified using the isolation forest algorithm, while the blank and moderately enhanced spectra were grouped through K-means clustering. The decision to form two clusters after removing the anomalous spectra is supported by visual inspection and a silhouette score analysis of multiple surface maps and concentrations (Figure S7). The silhouette score measures how well-separated and compact the clusters are, ranging from −1 to 1 where a higher score indicates better clustering.^53^ In this case, a consistently higher score was observed for two clusters (*K* = 2), suggesting that the data naturally formed two well-separated groups. Following clustering, only the spectra with moderate enhancement are retained for downstream semiquantification, as they provide the best balance between signal intensity and reproducibility.

### Semiquantification of Nanoplastics via Supervised Learning

After isolating the spectra with moderate enhancement through PCA and K-means clustering, all spectra from the surface mappings across the different PMMA nanoplastic concentrations were merged into a single data set. This data set was used to train a broad set of machine learning classification models (Table S4) using known concentrations as training labels within a stratified 5-fold cross-validation scheme. The objective is to develop a machine learning model that can classify samples into distinct concentration ranges based on the SERS spectra, thereby enabling semiquantification.

Two approaches were explored: classifiers trained directly on full, high-dimensional spectra or on PCA-transformed spectra computed inside each cross-validation fold. Each model was first benchmarked in assigning unseen spectra to the correct concentration class (Figure S8). After benchmarking, the top five models underwent hyperparameter tuning and were re-evaluated to select the strongest classification model. Figure S9 further displays how the performance metrics vary across individual train/test splits, offering a sense of stability across the folds.

With direct spectra, two models were highlighted: extremely randomized trees (ExtraTrees), an algorithm based on decision trees where the final prediction is made by majority vote, and a multilayer perceptron (MLP), a type of artificial neural network. Both achieved a cross-validated accuracy of 90%, although MLP displayed a better stability across folds (Figure S9). However, with PCA-transformed spectra, the strongest classifier was ExtraTrees, with a cross-validated accuracy of 95% and the highest stability among all tested models. This performance gain is consistent with previous work showing that ExtraTrees performs better on PCA-reduced Raman data.^54^ The confusion matrix of the ExtraTrees classifier shows precision, recall, and F1-score values of 96%, 95%, and 95%, respectively (Figure 2c).

Sampling, isolating, and characterizing nanoplastics pose significant challenges in complex environmental or biological matrices.^55^ As a result, quantitative measurements are often not practical. To advance nanoplastic monitoring and facilitate regulations, a threshold-based approach, consisting of determining whether nanoplastic concentrations reach predefined levels of concern, is more realistic for assessing ecological or human health risks.^56^ The semiquantitative approach demonstrated here reliably categorizes samples into discrete, meaningful concentration levels. However, translation to environmental water analysis will require dedicated validation in the presence of matrix constituents, such as dissolved organic matter, salts, and biomolecules, which may introduce matrix effects, compete for adsorption sites, and promote membrane fouling.

### Interpreting Model Predictions through Perturbation-Based Raman Shift Analysis

Machine learning models are powerful predictive tools for analytical applications, yet the rationale behind their predictions is often perceived as opaque.^57^ In SERS-based (bio)­analysis, model-interpretation strategies are particularly valuable to identify spectral peaks or regions critical for semiquantification and confirm that models rely on meaningful analyte-derived spectra.^47^ Two complementary strategies can be used: 1) feature importance metrics from interpretable models and 2) perturbation-based analysis of Raman shifts.

Tree-based classifiers such as ExtraTrees compute feature importance intrinsically, making them naturally interpretable. Our ExtraTrees classifier provides a ranked list of Raman shifts contributing most strongly to its decisions (Figure 3a), validating that the highest-importance shifts coincide with PMMA vibrational bands (Figure 3b; Table S2).

**Figure 3:**
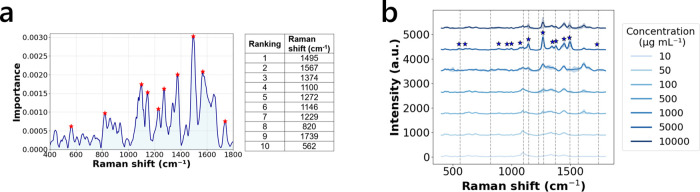
ExtraTrees feature importance aligned with PMMA vibrational bands. **a** Ranked feature importance obtained from the ExtraTrees classifier. Red stars mark the 10 top-ranked features listed on the inset Table. **b** Mean SERS spectra of the moderate-enhancement subset across PMMA concentrations (10 to 10,000 μg mL^–1^). Blue stars indicate PMMA vibrational modes (Table S2). Vertical dashed lines mark the Raman shifts of highest importance for semiquantification by ExtraTrees, confirming chemically meaningful spectral bands are prioritized.

A model-agnostic approach involves perturbing spectral features in the test set and assessing the resulting accuracy change, implemented through recursive permutation^58^ or local perturbation of individual spectral components.^59^ Spectral regions most essential for semiquantification appear as those producing the largest accuracy reductions.

We applied this perturbation-based approach to the three best-performing models identified earlier, ExtraTrees, MLP, and linear discriminant analysis (LDA), allowing direct comparison of how different algorithms rely on spectral information. To the best of our knowledge, this represents the first implementation of an interpretability algorithm in machine-learning-assisted SERS for the detection of nanoplastics, moving beyond conventional black-box models to provide a transparent, chemically validated analytical output.

The test set was perturbed with a Voigt profile sweeping the 400–1800 cm^–1^ Raman shift range (Supporting Information Video S2) simulating realistic distortions.^60^ Accuracy decreases were calculated relative to the unperturbed baseline via 5-fold cross-validation. Perturbation intensity and width were varied to probe model tolerance and expose groups of correlated spectral features that affect semiquantification only when perturbed collectively.

Results are summarized in Figures S10–S12. ExtraTrees produced the most interpretable results, highlighting only a few well-defined spectral regions, primarily around 1445 and 1100 cm^–1^. Most of these regions correspond to PMMA vibrational bands or contributions from the nanopaper background (Figure 4a,b). This confirms that semiquantification relies on analyte or substrate signatures rather than noise or artifacts and that the plasmonic membrane captures chemically meaningful spectral features from nanoplastics.

**Figure 4:**
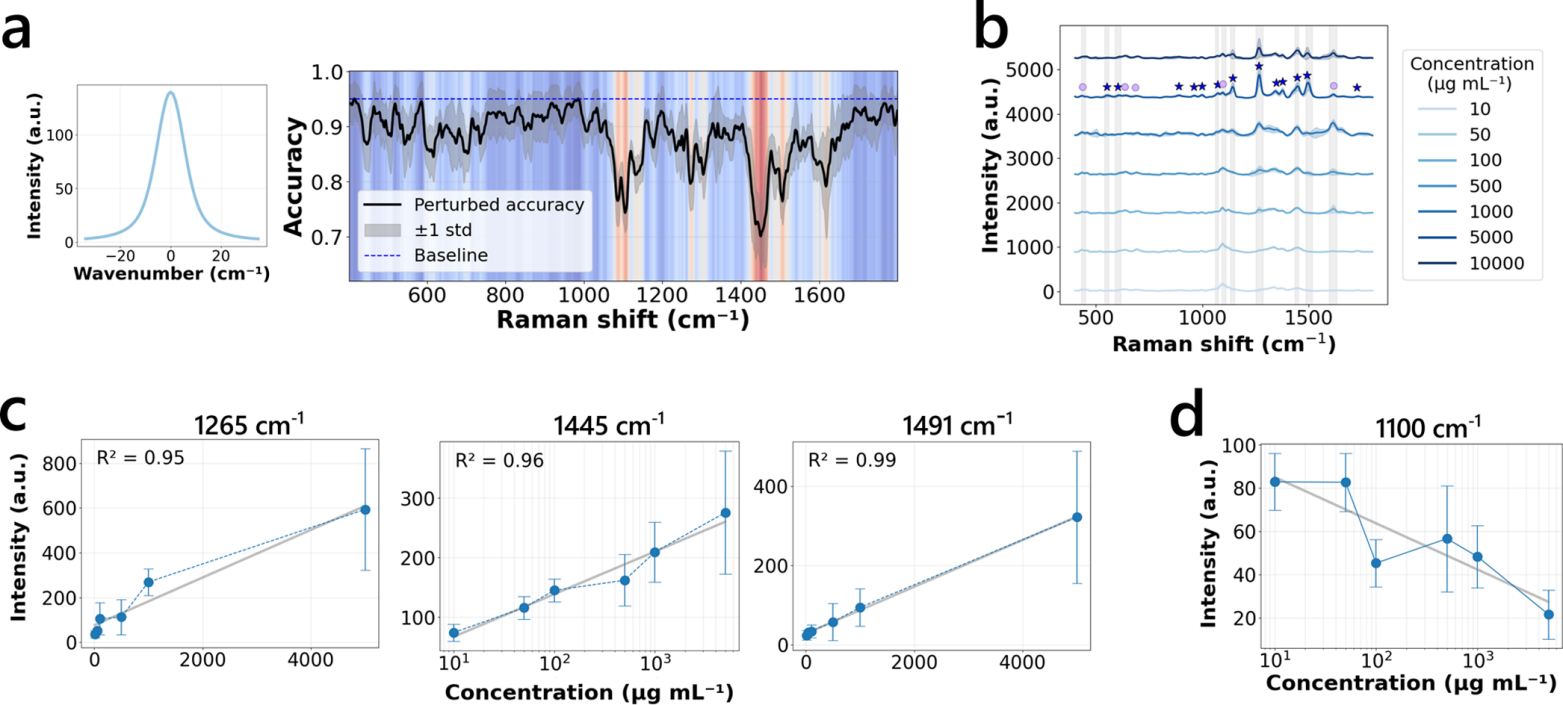
Interpretability and semiquantitative relevance of the spectra features identified by the ExtraTrees classifier. a (left) Example Voigt perturbation profile used to probe model robustness and (right) accuracy losses revealing the classifier relies on few well-defined spectral regions, primarily around 1445 and 1100 cm^–1^. **b** Mean SERS spectra of the moderate-enhancement subset across PMMA concentrations from 10 to 10,000 μg mL^–1^. Blue stars indicate PMMA vibrational modes; purple circles indicate vibrational modes from the nanopaper background; shaded regions mark the spectral regions of highest importance most consistent with PMMA or nanopaper contributions. **c** Intensity of key PMMA bands at 1265 (linear scale), 1445 (logarithmic scale), and 1491 cm^–1^ (the linear scale shows a linear fit with nanoplastic concentration), enabling semiquantitative analysis from 10 to 5000 μg mL^–1^. **d** The nanopaper-derived band at 1100 cm^–1^ (logarithmic scale) shows a progressive attenuation with nanoplastic loading due to surface screening.

Extending the classifier beyond the PMMA is feasible. Machine learning-assisted SERS has demonstrated the ability to discriminate nanoplastics based on their polymer-specific vibrational fingerprints across various systems, particularly when incorporating PCA.^10,61,62^ For example, PMMA exhibits a strong characteristic band associated with the methyl ester group around 1445 cm^–1^, while polystyrene is dominated by an aromatic ring mode around 1000 cm^–1^.^63^ Such spectral differences should allow multiclass discrimination provided the plasmonic membrane maintains signal enhancement across different polymer classes; moving toward multiplexed analysis will require both model retraining with multiclass data sets and validation that the membrane can simultaneously capture and enhance signals from other types of nanoplastics.

The MLP and LDA distribute importance across many regions, frequently located in valleys between peaks or even in featureless regions (Figure S13). Both models appear to depend on subtler shape-based attributes, like local curvatures, baseline structures, or compound correlations, although potentially concentration-correlated are not easily interpretable in spectroscopic terms. Both models also show substantial accuracy losses under even the weakest perturbation, indicating a reduced robustness to small distortions.

Overall, the ExtraTrees classifier demonstrates favorable performance not only for its accuracy but also because its decision process aligns with interpretable, chemically grounded spectral features and shows higher resistance to variations in the input SERS spectra.

### Using Key Raman Shifts for Nanoplastic Semiquantification via Calibration Curves

The Raman shifts highlighted as important for semiquantification by the ExtraTrees classifier also exhibit concentration-dependent intensity changes, enabling their use for semiquantitative analysis. The nanoplastic-derived bands selected through feature importance analysis show a rapid increase between 10 and 5000 μg mL^–1^ before approaching a plateau and then decreasing above 5000 μg mL^–1^ (Figure S14).

The plateau is consistent with a scenario in which most accessible enhancement sites become partially filled as nanoplastic concentration rises. Additional nanoplastics likely adsorb in regions that contribute less efficiently to SERS enhancement consistent with surface-limited adsorption processes, a well-known factor in semiquantification using SERS.^64^ The subsequent decrease at concentrations above 5000 μg mL^–1^ may originate due to a screening effect (Figure S15), in which dense nanoplastics physically obstruct the access to metal nanoparticles and increment light scattering, reducing overall enhancement. Although this effect limits nanoplastic semiquantification at high loadings, it is unlikely to affect practical applications that typically involve lower nanoplastic concentrations. Both the saturation and downturn at high concentrations agree with existing literature reporting other SERS-active membranes and different nanoplastics types.^10,19,22^

Within the 10–5000 μg mL^–1^ tested range, several PMMA bands exhibit intensity trends suitable for conventional calibration curve semiquantification. The bands centered at 1265, 1445, and 1491 cm^–1^, assigned to C–C–O stretching, CH_3_ bending, and CH_2_ bending modes of PMMA, show a linear relation with nanoplastic concentration (Figure 4c), either on a linear scale (1265 and 1491 cm^–1^) or on a logarithmic scale (1445 cm^–1^). This mode-specific sensitivity is a distinctive in SERS due to surface-selection rules and orientation-dependent enhancements.^65^

The corresponding linear calibration curves yield coefficients of determination (*R* ^2^) indicative of a suitable fit (Figure 4c**)**. We also estimated the limits of detection (LOD) for these calibration curves using the equations outlined in the Materials and Methods section.^66^ The resulting values are 0.03, 0.02, and 0.06 μg mL^–1^ for the models based on the bands at 1265, 1445, and 1491 cm^–1^, respectively. In addition, the calibration curve derived from the 1445 cm^–1^ band shows the lowest RSD %, with a value of 22.9 ± 8.5 (Table S5).

Recovery rates were calculated for the 1445 cm^–1^ and 1491 cm^–1^ curves, corresponding to the best LOD/RSD % and best *R* ^2^, respectively (Figure S16). The 1445 cm^–1^ curve is effective within 10–1000 μg mL^–1^ (80 to 125% recovery), as higher concentrations lead to increased standard errors and inaccuracies. The 1491 cm^–1^ calibration curve performs within 50–5000 μg mL^–1^ (96% to 112% recovery); however, estimations at lower concentrations yielded considerable errors due to an inadequate fit to the linear model.

Establishing a clear correlation between PMMA peak intensity and nanoplastic concentration was only possible after optimizing the data set through our machine learning pipeline, which included outlier removal and clustering of moderate-enhancement spectra (Figure S5). Nevertheless, due to the inherent residual variability in SERS detection of large analytes as nanoplastics, semiquantification using a fully machine learning approach, such as machine learning classifiers, can be considered a more robust and practical alternative to conventional univariate calibration curves.

The ExtraTrees classifier also assigns substantial importance to the 1100 cm^–1^ band, the most intense Raman feature of nanopaper within 400–1800 cm^–1^ (Figure S5a).^67^ Additional substrate-associated bands at 437 and 1610 cm^–1^ also appear with lower importance. These bands remain relatively stable or without a clear trend across nanoplastic concentrations, while the 1100 cm ^–1^ band shows a decrease as nanoplastic loading increases, see (Figures S17; 4d), suggesting a progressive screening of the nanopaper surface by nanoplastics.

Perturbation of the 1100 cm^–1^ band produces artificial intensity increases and disrupts the expected concentration-dependent decrease, reducing significantly semiquantification accuracy. This suggests that the substrate bands may serve as an internal reference, similar to ratiometric sensing approaches. A substrate with distinct and stable Raman signatures may therefore provide an internal benchmark that differentiates concentration-dependent changes from heterogeneity-induced variations in substrate or measurement conditions.^68^ Overall, the ExtraTrees model’s reliance on two concentration-dependent effects, growth of nanoplastic-related band intensity and subtle attenuation the substrate bands due to screening, may be contributing to its interpretability and robustness.

## Conclusions

This study demonstrates that integrating a low-cost (<$0.1USD, see Table S1 in the Supporting Information), easily fabricated plasmonic membrane with a fully automated machine learning pipeline enables reliable detection and semiquantification of nanoplastics, using PMMA spheres as a model analyte. The nanopaper scaffold facilitates direct analysis after immersion, eliminating complex sample preparation and providing a stable scaffold for the gold nanorod deposition. Despite substantial spectral heterogeneity in SERS mappings, the workflow combining PCA, automated outlier removal by isolation forests, and K-means clustering effectively manages this variability.

Semiquantification into distinct concentration ranges was evaluated using a comprehensive list of machine learning classifiers (Table S4). ExtraTrees demonstrated the best classification performance (95% accuracy and 96% precision) with interpretability and resistance to spectral variations. This approach demonstrates strong potential for threshold-based screening, showing potential for categorizing samples into meaningful categories (e.g., safe, risky, and harmful) applicable to filtered samples or, following matrix-specific validation, environmentally relevant samples.

Additionally, perturbation-based analysis confirmed that the ExtraTrees model relies primarily on chemically meaningful PMMA spectral bands as well as on nanopaper-related bands that behave as an internal reference. By bridging the gap between automated machine learning decision-making and physical spectral features, this framework represents, to the best of our knowledge, the first implementation of an interpretability algorithm for machine learning-assisted SERS-based nanoplastic detection. Future research can build on this approach to enhance reliability, user confidence, and regulatory acceptance for machine learning-based environmental monitoring systems.

The combination of analyte-specific and substrate-dependent spectral bands enhances the model stability against spectral distortions and supports PMMA nanoplastic semiquantification (a challenging nanoplastic rarely studied due to its weak Raman cross section compared to polystyrene) using peak-intensity calibration curves, with an estimated LOD of 0.02 μg mL^–1^. However, the fully machine learning-based ExtraTrees approach is a better alternative to univariate calibration curves for the semiquantification of large particles, such as nanoplastic, since this approach effectively addresses the inherent residual variability of the SERS signal and provide more robust predictions.

Overall, our automated end-to-end machine learning workflow for surface map processing delivers interpretable semiquantification results, highlight the key SERS peaks and vibrational modes driving the model’s predictions. This reduces the need for manual spectral analysis and band selection, facilitating rapid semiquantitative model development, advantageous for routine and user-friendly nanoplastic screening. Our findings underscore the potential of integrating simple SERS platforms with data-driven machine learning models, establishing a foundation for future field-deployable, scalable, and user-friendly nanoplastic screening tools.

Future work should focus on 1) validation in environmentally relevant matrices, where dissolved organic matter, salts, and biomolecular species may introduce matrix effects, competitive adsorption, and membrane fouling; 2) evaluating capture efficiency as a function of particle size, particularly in challenging regimes below 100 nm, and optimizing nanopaper porosity and surface chemistry to improve size-selective nanoplastic capture, 3) exploring alternative metal nanoparticles or detection strategies to boost sensitivity; and 4) extending the integrated membrane and machine learning workflow to multiplexed detection of other common nanoplastics, such as polystyrene, polyethylene, and polyethylene terephthalate, which will require validation of the polymer-level specificity of the membrane and retraining the model with multiclass data sets.

## Materials and Methods

### Chemicals and Materials

Silver nitrate (AgNO_3_), ascorbic acid (C_6_H_8_O_6_), hexadecyltrimethylammonium bromide (CTAB), and chloroauric acid (HAuCl_4_) were purchased from Sigma-Aldrich. Sodium borohydride was purchased from Karal (León, México). PMMA nano and submicron-plastics (500 nm) were purchased from Phosphorex (Boston, MA). Bacterial nanocellulose sheets were purchased from Nanonovin (Sari, Iran).

### Equipment

UV–vis absorption spectra were collected using a multimode microplate reader (Synergy H1, Biotek) with a 1 × 1 cm quartz and a 96 microplate for liquid and solid in-film measurements, respectively. Scanning electron microscopy (SEM) images were acquired using a high-resolution field emission electron microscope (JSM-7800F, JEOL). SERS spectra were collected using a Raman microscope (DXR3, ThermoFisher).

### Synthesis of Gold Nanorods (AuNRs)

The AuNRs were synthesized using a conventional seed-mediated growth method. Briefly, the seed solution was prepared by mixing 5 mL of HAuCl_4_ at 0.5 mM, 5 mL of CTAB at 0.2 M, and 0.6 mL of cold and fresh NaBH_4_ at 10 mM under vigorous stirring at room temperature. The solution was incubated for 45 min. The growth solution was prepared by mixing 100 mL of CTAB concentrated at 0.2 M, 3.5 mL of AgNO_3_ at 4 mM, 100 mL of HAuCl_4_ at 1 Mm, 1.8 mL of ascorbic acid at 78 mM, and 450 μL of the seed solution. The mixture was incubated for 3 h at room temperature. Finally, the AuNRs solution was washed once by centrifugation for 1h, at 4500 rpm, and resuspended in Milli-Q water until use. To assess interbatch reproducibility, three independent AuNR synthesis batches were prepared, and morphology metrics (i.e., length, width, and aspect ratio) were evaluated from the SEM images. Generally, the resulting AuNRs showed a length of 40 ± 7 nm and a width of 11 ± 1 nm.

### Fabrication of the plasmonic Membrane

The plasmonic membranes were fabricated by using an immersion method. Bacterial nanocellulose sheets of 2.5 × 2.5 cm were immersed in a solution of gold nanorods (AuNR) overnight under low agitation. This process allows for the immobilization of the AuNRs within the nanocellulose fibers. After immersion, the plasmonic membranes were washed three times with Milli-Q water to remove any unbound AuNRs. The membranes were then dried by placing them between two pieces of filter paper and applying pressure with two clamped glass slides. Finally, the plasmonic membranes were stored in a dark environment at room temperature, until needed.

The stability of the plasmonic membrane against nanoparticle leaching was evaluated by immersing the gold nanorod-functionalized nanopaper in Milli-Q water under gentle agitation (6 Hz at a linear motion). Samples were subjected to varying immersion periods spanning short-term (0.5, 5, 10, 30, and 60 min) and long-term (24 h) intervals. Membrane interbatch fabrication reproducibility was assessed by measuring absorbance spectra from 15 plasmonic membranes fabricated from three independent batches (5 membranes per batch).

### Preparation and Collection of Nanoplastic Samples

PMMA spheres of 500 nm were diluted in Milli-Q water to concentrations ranging from 10 to 10,000 μg mL^–1^ to generate a spiked-sample data set for the validation of the integrated plasmonic membrane and machine learning workflow. Plasmonic membrane disks (6 mm diameter) were cut and immersed in 0.5 mL of these spiked solutions for 30 min. The disks were then placed on a glass slide covered with aluminum foil and allowed to dry prior to SERS analysis.

### Raman and SERS Measurements

Raman and SERS spectra were recorded using a confocal Raman microscope (DXR3, ThermoFisher) equipped with a 20× objective lens and an excitation wavelength of 785 nm (≈10 mW), providing an effective lateral spot size of ∼1 μm, consistent with the diffraction-limited resolution for this configuration. Raman spectra of pure BNC and PMMA were collected using single point measurements with 60 s integration time and one accumulation. Membrane interbatch reproducibility of background signal was evaluated from blank plasmonic membranes using three membranes per batch and four randomly selected measurements points per membrane, collected using 1s integration and 3 accumulations.

The PMMA nanoplastic-spiked samples were analyzed using surface mappings, generally collecting an array of 6 × 6 over a scan area (≈5000 μm^2^) with 1s integration for each scanning point and 3 accumulations per point to balance speed, signal quality, and sample preservation. The scan areas were selected after confirming the presence of nanoplastics. Raman spectra were baseline-corrected using the I-Mod-Poly algorithm^69^ and smoothed using a Savitzky–Golay filter.

The limits of detection (LOD) reported for the peak-intensity calibration curves were estimated from the analytical response using the formula LOD = LOB +1.645σ_lowest concentration sample_, where the limit of blank (LOB) is defined as LOB = μ_blank_ + 1.645σ_blank_ and μ_blank_ represents the mean value of blank measurements.^66^ Here, μ and σ indicate intensity measurements obtained from the SERS spectra. The final LOB and LOD values in μg mL^–1^ are interpolated from the calibration curves.

### Declaration of Generative AI and AI-Assisted Technologies in the Writing Process

The authors used Nature Research Assistant and ChatGPT to improve the grammar, clarity, and readability. The initial draft, including its structure, content, and interpretation of results, was entirely composed by the authors. All suggestions from the AI tools were carefully revised and approved by the authors.

## Supplementary Material







## Notes

Membrane immersion stability; batch-to-batch reproducibility data; scanning electron microscopy images of bacterial nanocellulose hierarchical porosity and nanoplastic retention; additional SERS analysis of the plasmonic membrane; machine learning evaluation and results of perturbation analysis; concentration-dependent curves of Raman bands; recovery rate plots; cost analysis of the plasmonic membrane; peak assignments; and list of the evaluated machine learning classifiers (PDF)
Disposal of the plasmonic membrane via combustion (MP4)
Voigt profile sweeping data (MP4)
