# Supplementary material for: Integrated SERS and Machine Learning Workflow for Nanoplastic Detection on a Plasmonic Membrane
Source: Anal Chem. 2026 Jul 8;98(28):20748–60. doi: 10.1021/acs.analchem.6c00075 (PMC13393082; doi:10.1021/acs.analchem.6c00075)
Supplement: Supplementary file 1 [file ac6c00075_si_001.pdf]

Supporting Information

Integrated SERS and Machine Learning Workflow for Nanoplastic Detection on a Plasmonic Membrane

Amauri Horta-Velázquez[1,2], Erika Rodríguez-Sevilla[1], Angelica Hernandez-Rayas[3], Miguel A. Vallejo[3] and Eden Morales-Narváez[2*]*

[1] Centro de Investigaciones en Óptica (CIO), A. C., Loma del Bosque 115, Lomas del Campestre, León, 37150, Guanajuato, México

[2] Universidad Nacional Autónoma de México, Centro de Física Aplicada y Tecnología Avanzada, Biophotonic Nanosensors Laboratory, Boulevard Juriquilla 3001, 76230, Querétaro, México

[3] División de Ciencias e Ingenierías, Universidad de Guanajuato, Colonia Lomas del Campestre, Campus León, Loma del Bosque 103, León, 37150, Guanajuato, México

*E-mails: amauri.vel@gmail.com, eden@fata.unam.mx

Table of Contents

Figure S1. Evaluation of plasmonic membrane during aqueous immersion..3

Figure S2. Batch-to-batch reproducibility of gold nanorod synthesis and plasmonic membrane fabrication3

Figure S3. Physical entrapment of 500 nm PMMA nanoplastics within the membrane. 4

Figure S4. Visual estimation of accessible pore sizes in bacterial nanocellulose nanopaper. 4

Figure S5. Raman and SERS analysis of PMMA nanoplastics on the plasmonic membrane......................4

Figure S6. Spectra variability observed on nanoplastic quantification via surface maps. 5

Figure S7. Silhouette score analysis used to determine the optimal number of clusters 5

Figure S9. Variation of classification performance across cross-validation folds under two training strategies. 6

Figure S10. Perturbation analysis of the ExtraTrees classifier 7

Figure S11. Perturbation analysis of the multilayer perceptron (MLP) classifier. 8

Figure S12. Perturbation analysis of the Linear Discriminant Analysis (LDA) classifier. 9

Figure S13. Spectral regions highlighted by MLP and LDA during the perturbation analysis 9

Figure S14. Concentration-dependent behavior of the nanoplastic-originated Raman bands identified as most important by the ExtraTrees classifier...10

Figure S15. Screening effect at high nanoplastic loading on the plasmonic membrane 10

Figure S16. Recovery rate (%) as a function of PMMA concentration for the 1445 cm^{-1} and 1491 cm^{-1} calibration models...10

Figure S17. Behavior of substrate-derived Raman bands across different nanoplastic concentrations.....11

Table S1. Itemized cost of the plasmonic membrane...11

Table S2. Raman peak assignments of PMMA in the 400-1800 cm^{-1} range 11

Table S3. Raman peak assignments of bacterial nanocellulose in the 400-1800 cm^{-1} range. 11

Table S4. Machine-learning classifiers evaluated for nanoplastic concentration prediction from SERS spectra. 12
Table S5. Coefficients of variation for the calibration curves used in the quantification of nanoplastics. ...12

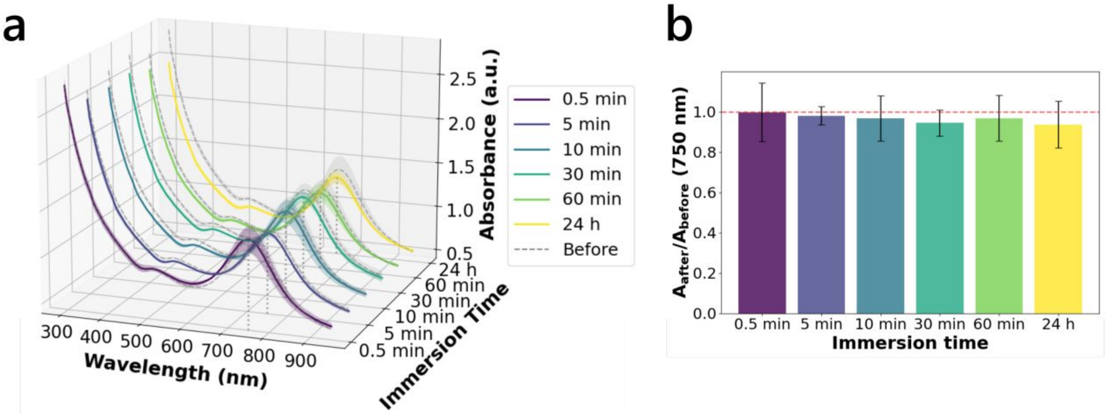

Figure S1. Evaluation of plasmonic membrane during aqueous immersion. (a) Absorption spectra of the gold nanorod-functionalized nanopaper before (gray dotted lines) and after (colored lines) immersion in water for different time intervals (ranging from 0.5 minutes to 24 hours) under gentle agitation. (b) Ratio of the absorbance at the localized surface plasmon resonance (LSPR) peak after immersion relative to the initial absorbance, demonstrating stable nanoparticle retention and the absence of significant detachment.

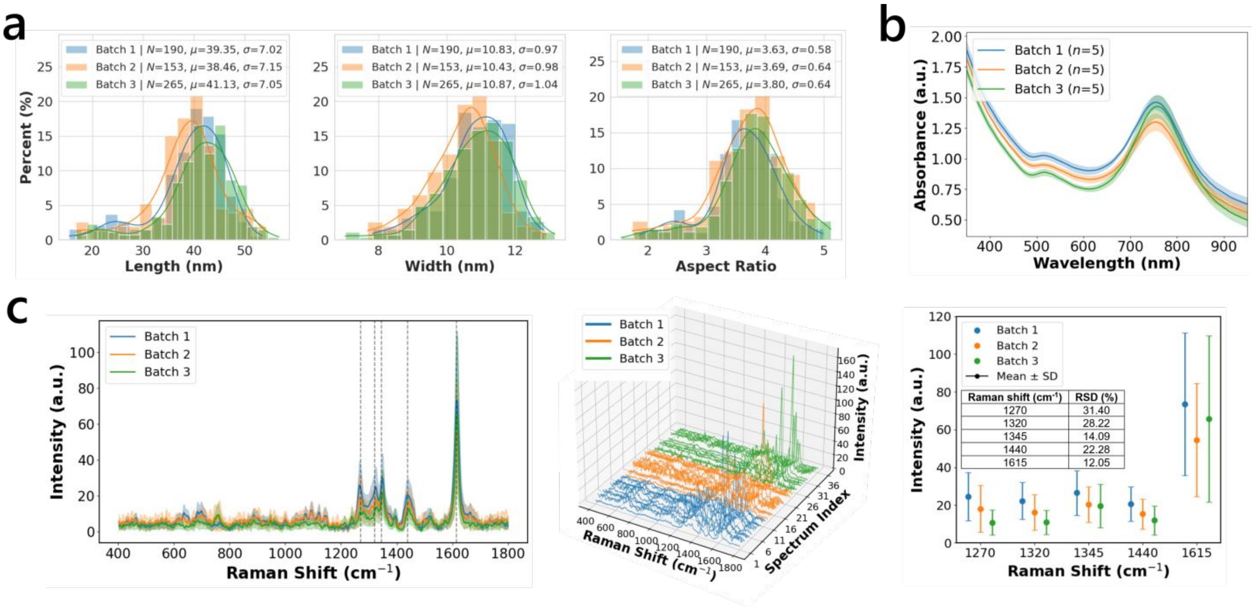

Figure S2. Batch-to-batch reproducibility of gold nanorod synthesis and plasmonic membrane fabrication. a) Histograms of gold nanorod length, width, and aspect ratio for three independent synthesis batches. b) Mean absorbance spectra of plasmonic membranes fabricated from each gold nanorod batch (n= 5 membranes per batch; shaded areas represent ± standard deviation, SD). The spectra show similar and overlapping profiles, suggesting comparable gold nanorod incorporation and preserved optical properties c) (*Left*) Mean background SERS spectra of blank plasmonic membranes from three fabrication batches (3 membranes per batch, 4 random measurements per membrane; shaded areas represent ± SD). Vertical dashed lines indicate the five most intense background bands. *(Center)* Three-dimensional representation of all 36 background spectra used for inter-batch reproducibility assessment. *(Right)* Mean intensity ± SD of the five main membrane background bands for each batch. Inset: Inter-batch relative standard deviation (RSD).

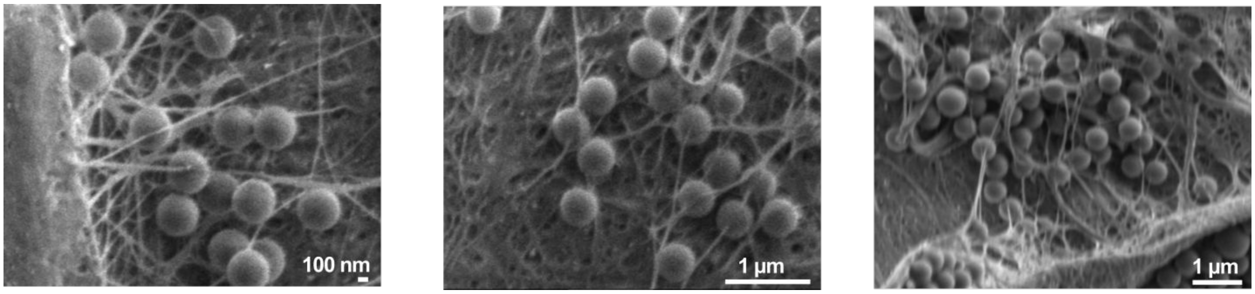

Figure S3. Physical entrapment of 500 nm PMMA nanoplastics within the membrane. Scanning electron microscopy (SEM) images illustrating the retention of PMMA nanoplastics within accessible voids of the nanopaper structure.

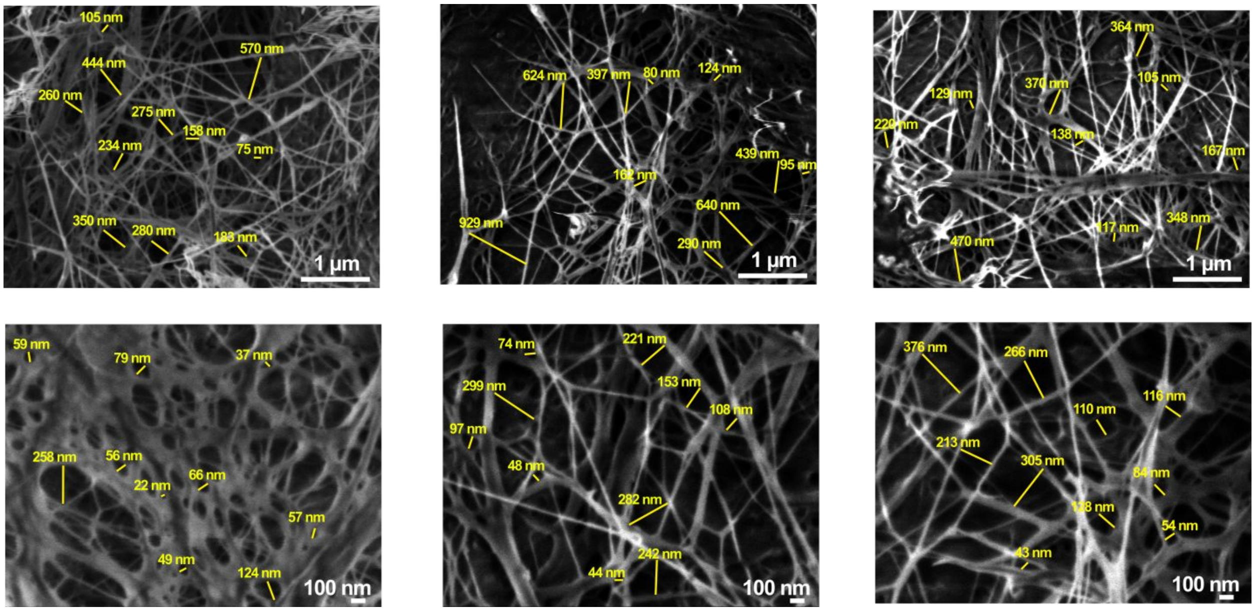

Figure S4. Visual estimation of accessible pore sizes in bacterial nanocellulose nanopaper. SEM images of nanopaper highlighting representative pores visible with the entangled nanofibrillar network. The annotation shows accessible pores spanning approximately 40 nm to 1 µm, in agreement with literature reports. These images provide only a visual estimate intended to illustrate accessible voids for nanoplastic entrapment; smaller and larger pores may be present but not resolved under imaging conditions.

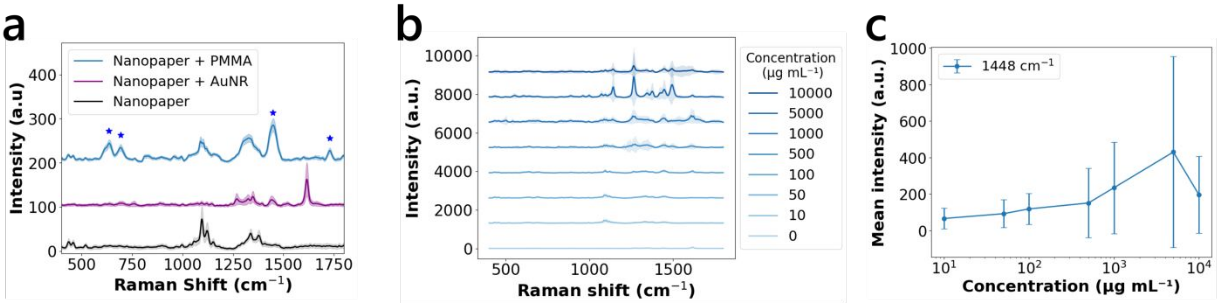

Figure S5. Raman and SERS analysis of PMMA nanoplastics on the plasmonic membrane. a) Raman spectra of pure nanopaper, nanopaper with gold nanorods (plasmonic membrane), and PMMA nanoplastic deposited on nanopaper (non-SERS conditions). The characteristic peaks of PMMA at 635, 693, 1268, 1448, and 1730 cm^{-1} are marked with blue stars. b) Average SERS spectra of PMMA nanoplastics (500 nm) at different concentrations measured at random points across the plasmonic membrane and c) mean intensity at 1448 cm^{-1} as a function of nanoplastic concentration.

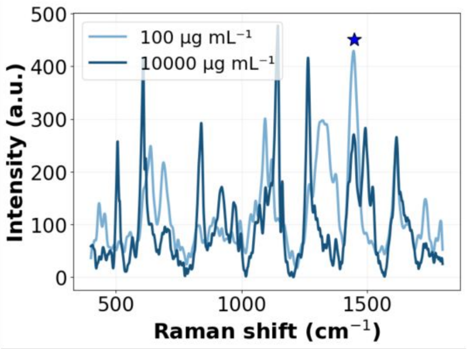

Figure S6. Spectra variability observed on nanoplastic quantification via surface maps. A low-concentration sample may produce a spectrum with intensity comparable to that of two orders of magnitude higher at some points, illustrating a spatial heterogeneity that limits conventional intensity-based quantification based on random averaging.

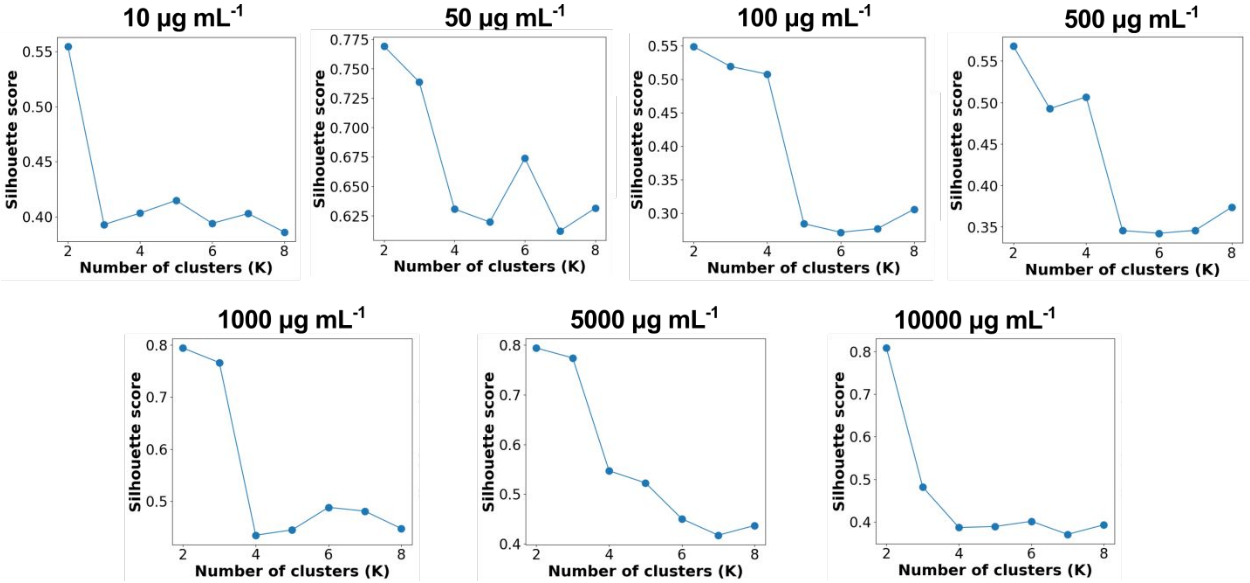

Figure S7. Silhouette score analysis used to determine the optimal number of clusters. Higher scores indicate better separation and compactness. Across multiple surface maps at different concentrations, the silhouette score consistently favors K=2, supporting the separation of blank and moderately enhanced spectra. Increasing the number of clusters only creates smaller, less meaningful subgroups.

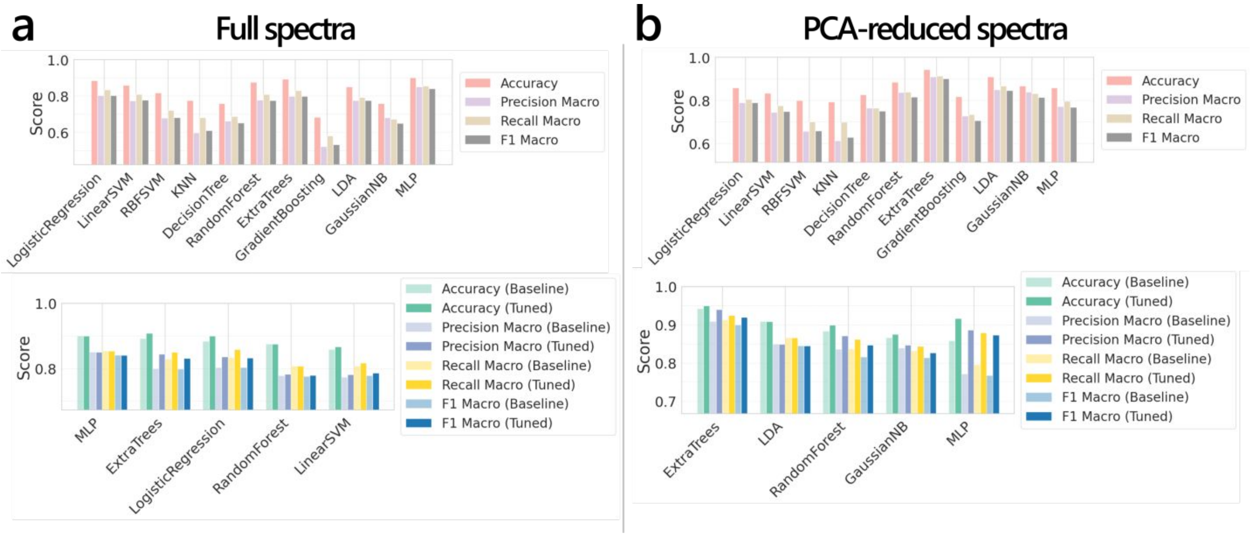

Figure S8. Benchmarking and optimization of different machine learning classification models. a. Classifiers trained and tested with full, high-dimensional SERS spectra. **b.** Classifiers trained and tested low-dimensional PCA-reduced SERS spectra. (Top) Cross-validated performance of all baseline models in classifying spectra by concentration. (Bottom) Comparison between baseline and optimized versions of the top five models after hyperparameter tuning, used to select the best-performing classifier.

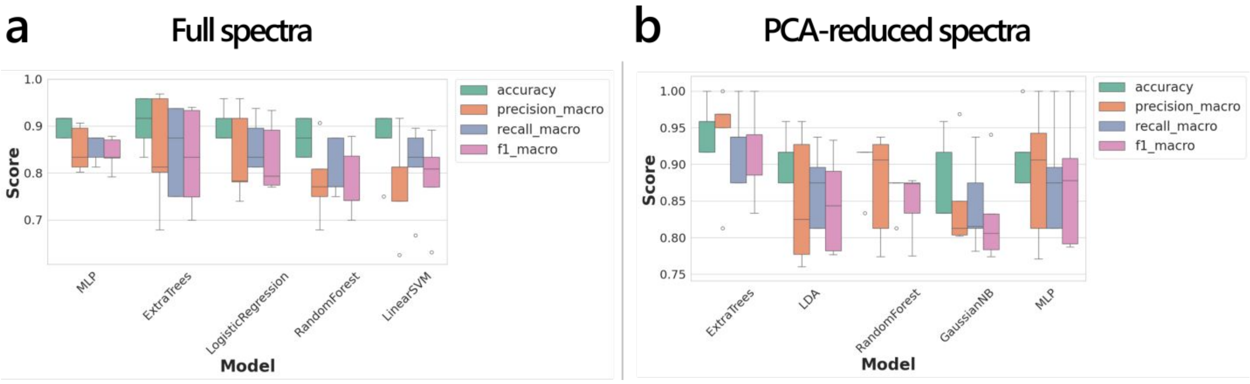

Figure S9. Variation of classification performance across cross-validation folds under two training strategies. a. using the full SERS spectra and **b.** applying PCA within each fold before training. With PCA-transformed spectra, ExtraTrees achieved the highest accuracy (95%) and displayed the most sable performance among different train/test splits.

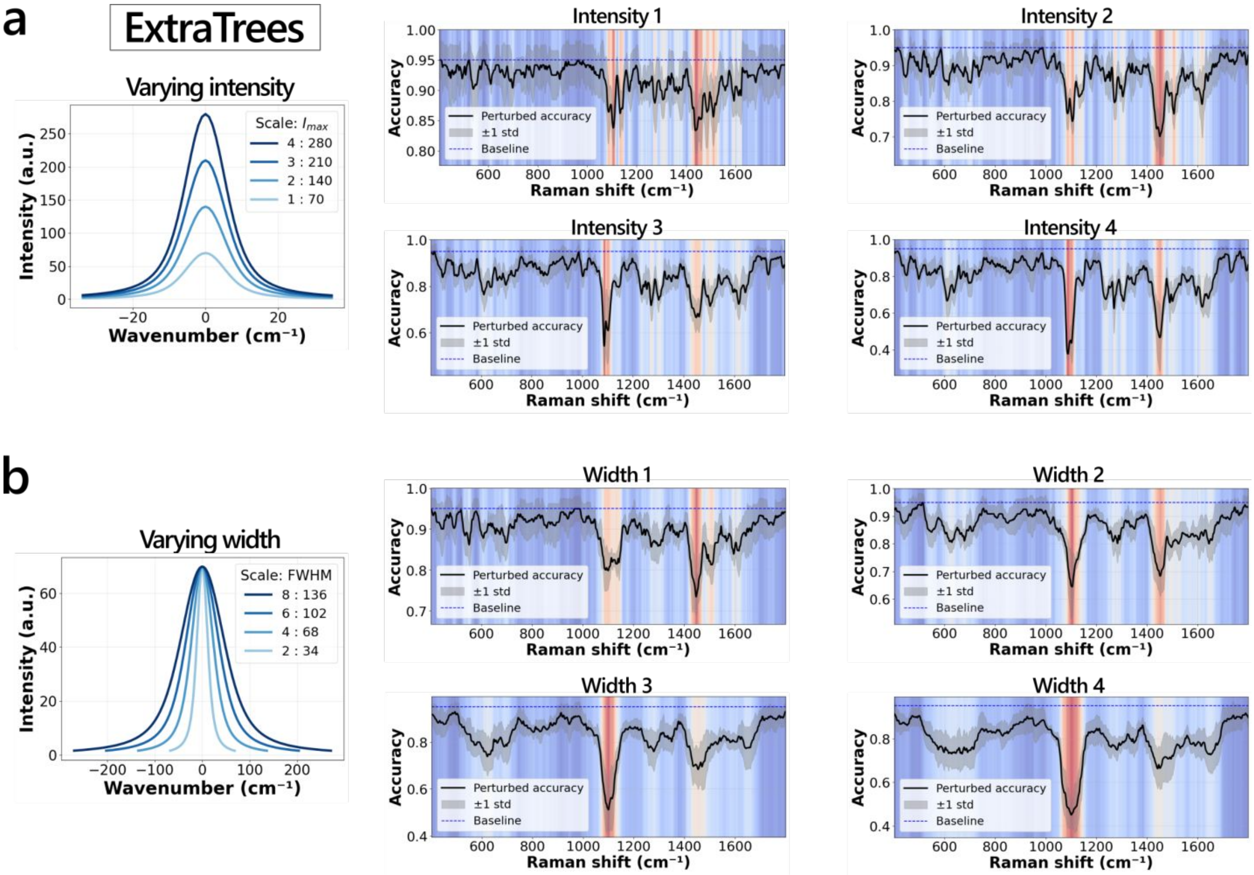

Figure S10. Perturbation analysis of the ExtraTrees classifier. The plots illustrate how classification accuracy changes when Voigt-shaped perturbations of varying **a.** intensity and **b.** width are applied across the Raman shift range. The classifier prioritizes a few well-defined regions, primarily around 1445 and 1100 cm^{-1}, confirming that the model relies on chemically meaningful features.

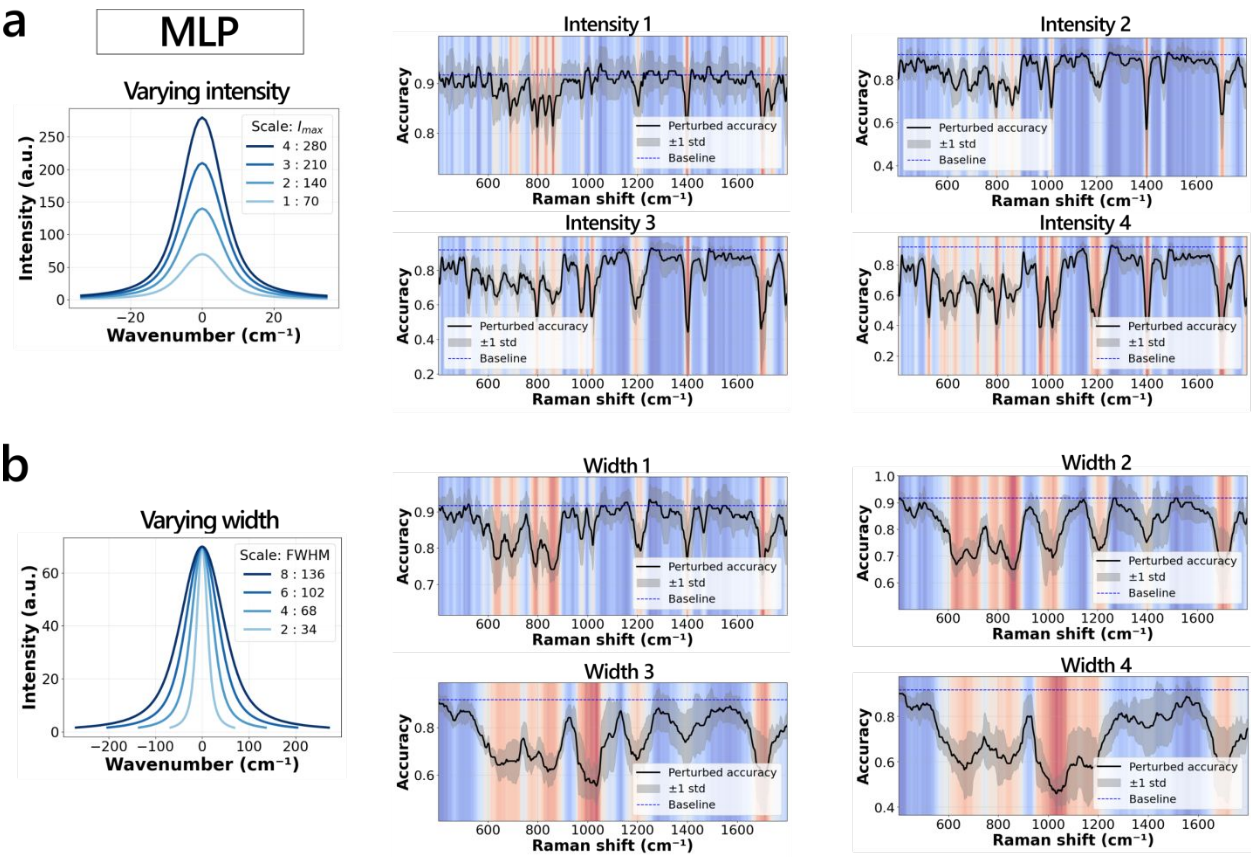

Figure S11. Perturbation analysis of the multilayer perceptron (MLP) classifier. The plots illustrate how classification accuracy changes when Voigt-shaped perturbations of varying **a.** intensity and **b.** width are applied across the Raman shift range. In both scenarios, the MLP distributes importance across several spectral region, many of them not associated to peak-centered features, suggesting that the model depends more on shape-based attributes, such as local curvatures and valleys. The model also experiences substantial accuracy losses even with minimal perturbation, showing that the model is highly sensitive to small distortions. Both facts lead to a reduced interpretability and lower robustness.

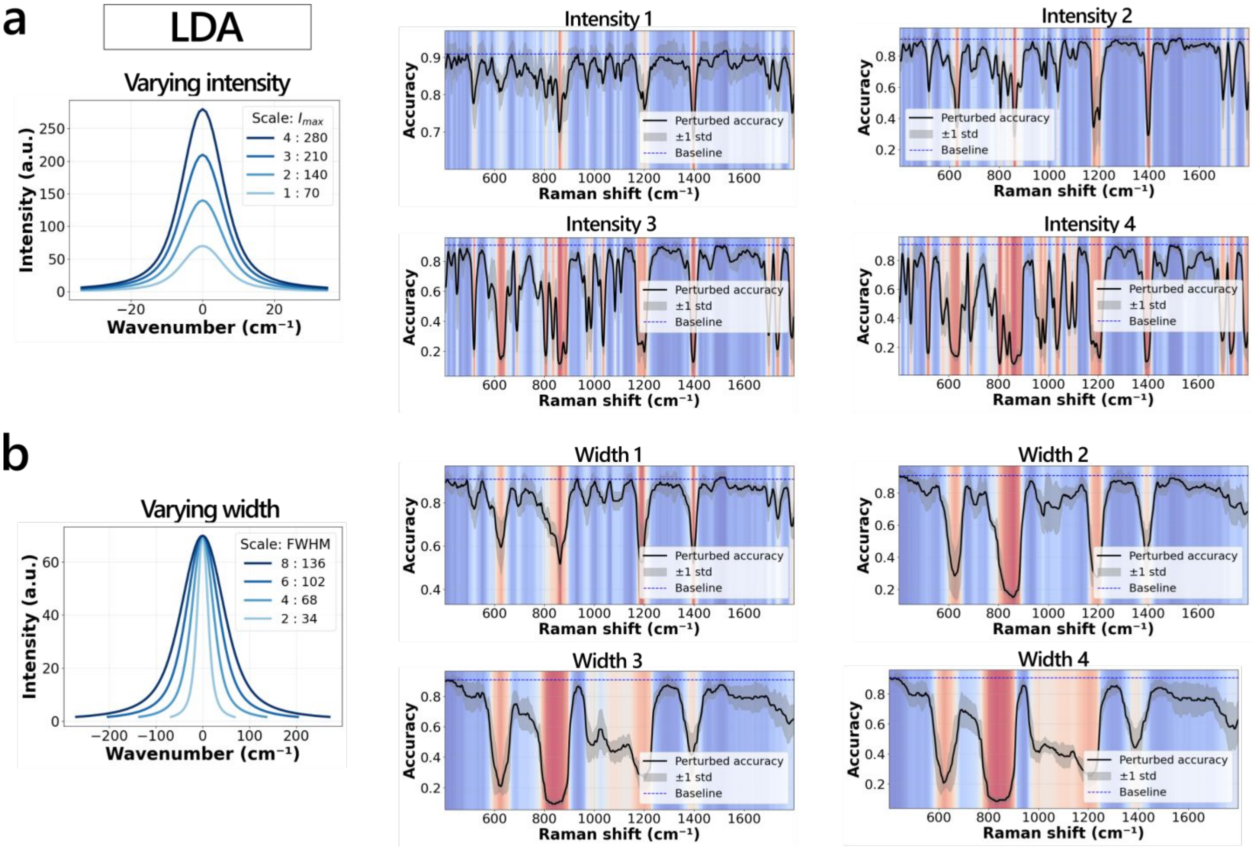

Figure S12. Perturbation analysis of the Linear Discriminant Analysis (LDA) classifier. The plots illustrate how classification accuracy changes when Voigt-shaped perturbations of varying **a.** intensity and **b.** width are applied across the Raman shift range. LDA shows widespread sensitivity across many regions of the spectrum, including valleys and featureless zones, such as MLP. Accuracy also declines rapidly even under the weakest perturbations, indicating limited robustness.

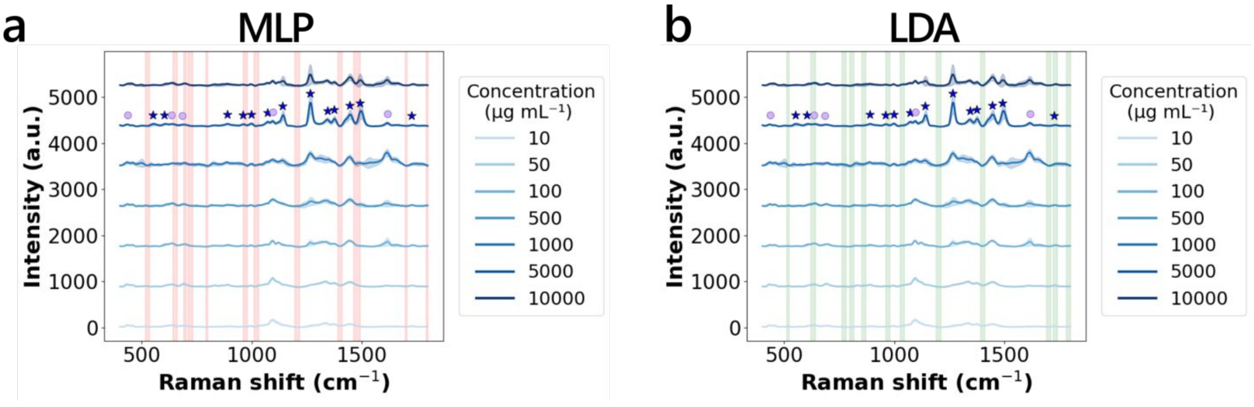

Figure S13. Spectral regions highlighted by MLP and LDA during the perturbation analysis. Mean SERS spectra of PMMA nanoplastics (10–10000 μg mL⁻¹) are shown with blue stars marking PMMA vibrational bands and purple circles indicating signals originating from the nanopaper substrate. The shaded vertical regions denote the Raman shifts to which to **a.** the MLP and **b.** the LDA classifiers are more sensitive. Unlike ExtraTrees, MLP and LDA distribute importance over many regions, including valleys and featureless regions. This suggest that both models rely on complex, less interpretable features, like local curvatures and baselines.

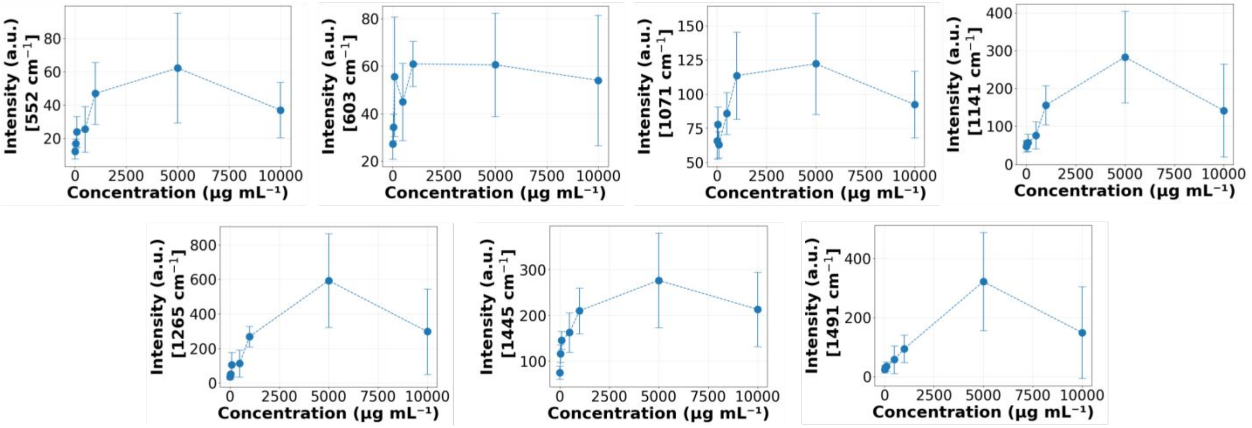

Figure S14. Concentration-dependent behavior of the nanoplastic-originated Raman bands identified as most important by the ExtraTrees classifier. Intensity trends for key PMMA vibrational bands exhibit a pronounced rise from 10 to 5000 μg mL^{-1}, followed by a plateau and a subsequent decrease at higher concentrations. The signal saturation aligns with surface-limited adsorption on the plasmonic membrane, where enhancement sites become increasingly occupied. The decrease after 5000 μg mL^{-1} likely reflects screening and scattering effects cause by nanoplastic coverage at high loadings, which reduces overall enhancement.

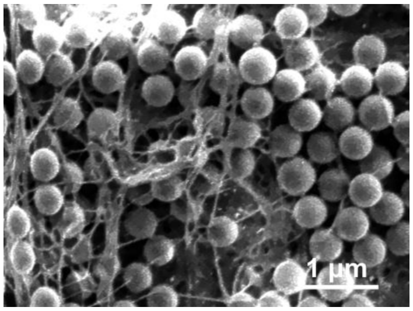

Figure S15. Screening effect at high nanoplastic loading on the plasmonic membrane. SEM image of the plasmonic membrane after immersion in a 10,000 μg mL^{-1} PMMA nanoplastic sample, showing a dense particle accumulation on the surface.

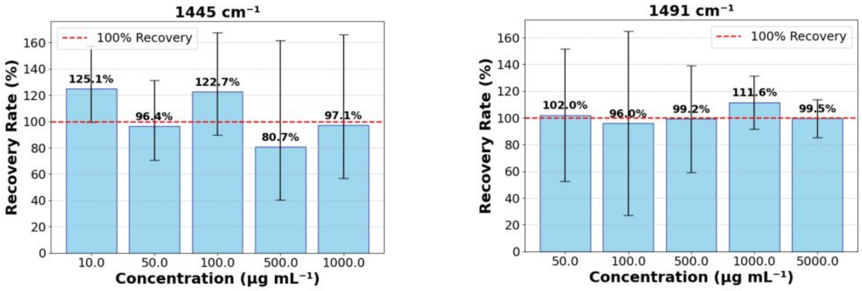

Figure S16. Recovery rate (%) as a function of PMMA concentration for the 1445 cm^{-1} and 1491 cm^{-1} calibration models. The 1445 cm^{-1} peak provides optimal accuracy for low concentrations (10–1000 μg ml^{-1}), since variability in intensity measurements at higher concentrations lead to significant measurement discrepancies. Meanwhile, the 1491 cm^{-1} peak is more effective for a higher concentration range (50–5000 μg ml^{-1}) due to its superior linear fit at those levels.

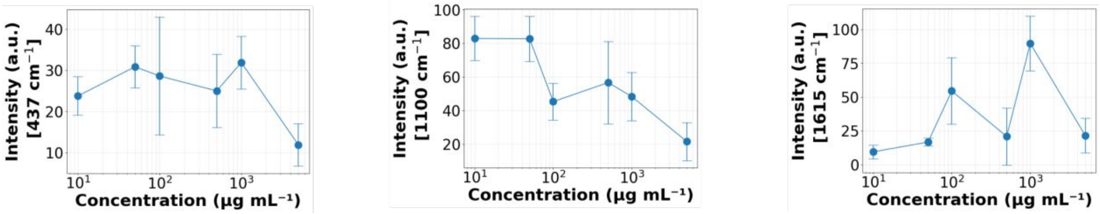

Figure S17. Behavior of substrate-derived Raman bands across different nanoplastic concentrations. Intensity trends for the nanopaper-associated bands at 437, 1100, and 1615 cm^{-1} as a function of PMMA nanoplastic concentration (10 to 5000 μg mL^{-1}). The 437 and 1615 cm^{-1} remain relatively stable or without a clear trending. Meanwhile, the band at 1100 cm^{-1} show a progressive decrease with increasing nanoplastic loading, consistent with screening of the nanopaper surface by adsorbed nanoplastics.

Table S1. Itemized cost of the plasmonic membrane.

**Material**	**Supplier**	**Cost (USD)**
BC	Nano Novin Polymer Co.	0.50
HAuCl4	Sigma-Aldrich	0.44
AgNO3	Sigma-Aldrich	0.001
NaBH4	Sigma-Aldrich	0.00006
Ascorbic acid	Sigma-Aldrich	0.0008
CTAB	Sigma-Aldrich	0.2
	**Total per sheet**	**1.1***
	**Plasmonic membrane**	**0.12**

**Note: Nine plasmonic membrane disks per 2.5 x 2.5 cm sheet*

Table S2. Raman peak assignments of PMMA in the 400-1800 cm^{-1} range

Raman shift (cm^{-1})	Vibrational mode	Reference
552	δ(C-C-C) skeletal	1–3
603	v(C-C-O)	1,2
625	v(C-C-O)	4
693	v(C-O-C)	4
888	v(CH_2)	2
960	δ(C-C)	5
998	γ(O-CH_3)	1,2
1071	δ(C-C) skeletal	2,5
1141	v(C-O-C)	1,3
1265	v(C-C-O-) or C-O stretch	1,3
1345	τ(CH_2)	5
1375	δ(C-H) of α(CH_3)	1
1446	δ(C-H) of CH_3	1–3,5
1491	δ(CH_2)	1,3
1725	δ(C=O) of (C-COO)	2,3,5

v= stretching, δ= bending, τ = torsion, γ = rocking

Table S3. Raman peak assignments of bacterial nanocellulose in the 400-1800 cm^{-1} range.

Raman shift (cm^{-1})	Vibrational mode	Reference
437	δ skeletal	6,7
635	δ(CH_2)	8

685	δ(O-H)	[9]
1100	ν(C-O-C)	[6,10,11]
1270	γ(H-C-C)	[6]
1320	*γ*(CH_2)	[6,9]
1380	δ(C-H)	[6,9]
1430	*δ*(CH_2)	[9,11]
1615*	*δ*(C-O-O)	[12,13]

ν= stretching, δ= bending, τ = torsion, γ = wagging

**Note: Plausibly attributed to carboxylate groups formed by mild surface oxidation of nanocellulose during AuNR immobilization.* [14,15]

Table S4. Machine-learning classifiers evaluated for nanoplastic concentration prediction from SERS spectra.

No.	Model (short name)	Algorithm/Family
1	LogisticRegression	Multinomial logistic regression (linear classifier)
2	LinearSVM	Linear support-vector machine
3	RBFSVM	Non-linear support-vector machine with RBF kernel
4	KNN	k-nearest neighbors classifier
5	DecisionTree	Single decision tree
6	RandomForest	Random forest (ensemble of decision trees)
7	ExtraTrees	Extremely randomized trees (ensemble of decision trees)
8	GradientBoosting	Gradient-boosted decision trees
9	LDA	Linear discriminant analysis
10	GaussianNB	Naïve Bayes with Gaussian likelihoods
11	MLP	Feed-forward neural network (multilayer perceptron)

Table S5. Coefficients of variation for the calibration curves used in the quantification of nanoplastics.

Raman shift (cm-1)	μ_{CV} (%)	σ_{CV} (%)
1265	45	22
1445	22.9	8.5
1491	49	20

Supplementary Video S1. Combustion-based disposal of the gold nanorod-functionalized nanopaper plasmonic membrane. The video shows a used plasmonic membrane, consisting of a bacterial nanocellulose (nanopaper) functionalized with gold nanorods, being disposed of by direct ignition.

Supplementary Video S2. Voigt profile sweep across the SERS spectral window for perturbation-based model interpretability analysis. The video shows a Voigt perturbation profile translating sequentially across the 400–1800 cm^{-1} Raman shift range, simulating realistic spectral distortions applied to the test set of SERS surface-map spectra. This sweep was used to assess the sensitivity of three machine learning classifiers, ExtraTrees, a Multilayer Perceptron (MLP), and Linear Discriminant Analysis (LDA), to localized spectral perturbations.

Supporting References

(1) Willis, H. A.; Zichy, V. J. I.; Hendra, P. J. The Laser-Raman and Infra-Red Spectra of Poly(Methyl Methacrylate). *Polymer* **1969**, *10*, 737–746. https://doi.org/10.1016/0032-3861(69)90101-3.

(2) Xingsheng, X.; Hai, M.; Qijing, Z.; Yunsheng, Z. Properties of Raman Spectra and Laser-Induced Birefringence in Polymethyl Methacrylate Optical Fibres. *J. Opt. A: Pure Appl. Opt.* **2002**, *4* (3), 237–242. https://doi.org/10.1088/1464-4258/4/3/303.

(3) Dybal, J.; Krimm, S. Normal-Mode Analysis of Infrared and Raman Spectra of Crystalline Isotactic Poly(Methyl Methacrylate). *Macromolecules* **1990**, *23* (5), 1301–1308. https://doi.org/10.1021/ma00207a013.

(4) Xu, G.; Cheng, H.; Jones, R.; Feng, Y.; Gong, K.; Li, K.; Fang, X.; Tahir, M. A.; Valev, V. K.; Zhang, L. Surface-Enhanced Raman Spectroscopy Facilitates the Detection of Microplastics <1 Mm in the Environment. *Environ. Sci. Technol.* **2020**, *54* (24), 15594–15603. https://doi.org/10.1021/acs.est.0c02317.
(5) Matamoros-Ambrocio, M.; Sánchez-Mora, E.; Gómez-Barojas, E. Surface-Enhanced Raman Scattering (SERS) Substrates Based on Ag-Nanoparticles and Ag-Nanoparticles/Poly (Methyl Methacrylate) Composites. *Polymers* **2023**, *15* (12), 2624. https://doi.org/10.3390/polym15122624.
(6) Wiley, J. H.; Atalla, R. H. Band Assignments in the Raman Spectra of Celluloses. *Carbohydrate Research* **1987**, *160*, 113–129. https://doi.org/10.1016/0008-6215(87)80306-3.
(7) Kim, S. H.; Lee, C. M.; Kafle, K. Characterization of Crystalline Cellulose in Biomass: Basic Principles, Applications, and Limitations of XRD, NMR, IR, Raman, and SFG. *Korean J. Chem. Eng.* **2013**, *30* (12), 2127–2141. https://doi.org/10.1007/s11814-013-0162-0.
(8) Benali, Y.; Mabrouki, N.; Agougui, H.; Jabli, M.; Majdoub, H.; Predoi, D.; Ciobanu, S.; Iconaru, S. L.; Ţălu, Ş.; Boughzala, K. A New Porous Composite Hydroxyapatite/Chitosan/Microcrystalline-Cellulose: Synthesis, Characterization and Application to the Adsorption of Eriochrome Black T. *Polym. Bull.* **2024**, *81* (18), 16875–16902. https://doi.org/10.1007/s00289-024-05496-3.
(9) Salem, K. S.; Kasera, N. K.; Rahman, Md. A.; Jameel, H.; Habibi, Y.; Eichhorn, S. J.; French, A. D.; Pal, L.; Lucia, L. A. Comparison and Assessment of Methods for Cellulose Crystallinity Determination. *Chem. Soc. Rev.* **2023**, *52* (18), 6417–6446. https://doi.org/10.1039/D2CS00569G.
(10) Boyaci, I. H.; Temiz, H. T.; Geniş, H. E.; Acar Soykut, E.; Yazgan, N. N.; Güven, B.; Uysal, R. S.; Bozkurt, A. G.; İlaslan, K.; Torun, O.; Dudak Şeker, F. C. Dispersive and FT-Raman Spectroscopic Methods in Food Analysis. *RSC Adv.* **2015**, *5* (70), 56606–56624. https://doi.org/10.1039/C4RA12463D.
(11) Agarwal, U. P. Analysis of Cellulose and Lignocellulose Materials by Raman Spectroscopy: A Review of the Current Status. *Molecules* **2019**, *24* (9), 1659. https://doi.org/10.3390/molecules24091659.
(12) Matsuki, S.; Kayano, H.; Takada, J.; Kono, H.; Fujisawa, S.; Saito, T.; Isogai, A. Nanocellulose Production via One-Pot Formation of C2 and C3 Carboxylate Groups Using Highly Concentrated NaClO Aqueous Solution. *ACS Sustainable Chem. Eng.* **2020**, *8* (48), 17800–17806. https://doi.org/10.1021/acssuschemeng.0c06515.
(13) Kaya, M. Characterization of TEMPO-Oxidized Cellulose Nanofiber From Biowaste and Its Influence on Molecular Behavior of Fluorescent Rhodamine B Dye in Aqueous Suspensions. *J Fluoresc* **2024**, *35* (6), 4053–4063. https://doi.org/10.1007/s10895-024-03824-4.
(14) Pandeirada, C. O.; Boulos, S.; Nyström, L. Oxidized Polysaccharides: A Review on Structural Insights Using MS-Based Approaches. *Carbohydrate Polymers* **2025**, *367*, 123946. https://doi.org/10.1016/j.carbpol.2025.123946.
(15) Tsunoyama, H.; Sakurai, H.; Negishi, Y.; Tsukuda, T. Size-Specific Catalytic Activity of Polymer-Stabilized Gold Nanoclusters for Aerobic Alcohol Oxidation in Water. *J. Am. Chem. Soc.* **2005**, *127* (26), 9374–9375. https://doi.org/10.1021/ja052161e.
